# Proximity labelling suggests association of the nonhost receptor PSS1 protein with enzymes of multiple defense pathways in Arabidopsis

**DOI:** 10.3389/fpls.2025.1701640

**Published:** 2026-03-05

**Authors:** Oluwatoyosi F. Akintayo, Christian Montes Serey, Justin W. Walley, Madan K. Bhattacharyya

**Affiliations:** 1 Interdepartmental Plant Biology, Iowa State University, Ames, IA, United States; 2 Department of Agronomy, Iowa State University, Ames, IA, United States; 3 Department of Plant Pathology, Entomology and Microbiology, Iowa State University, Ames, IA, United States

**Keywords:** Arabidopsis, mass spectrometry, miniTurbo, nonhost resistance, *Phytophthora sojae*, protein interactome, proximity labeling, PSS1

## Abstract

**Introduction:**

*PSS1* encoding a glycine-rich integral plasma membrane protein plays a pivotal role in nonhost resistance of Arabidopsis against the soybean pathogens, *Phytophthora sojae* and *Fusarium virguliforme*. Notably, *P. sojae* fails to penetrate the wildtype Col-0 ecotype but not the pss1 mutant. To elucidate the molecular basis of *PSS1*-mediated nonhost immunity, we employed a miniTurbo-based proximity labeling approach.

**Methods:**

The miniTurbo fused to either wild-type PSS1 or its mutant variant, PSS1^G119D^, and expressed in the *pss1* knockout mutant background. Seedlings were challenged with or without *P. sojae* zoospores and biotinylated proteins were isolated for mass spectrometry analysis. Col-0 and PSS1^G119D^ lines served as controls to validate labeling specificity and biological relevance.

**Results:**

Prior to infection, spatial proximity between PSS1 and the plasma membrane H(^+^)-ATPase 6 transporter was detected; however, this association was not observed following *P. sojae* challenge. Several candidate PSS1-associated proteins were predominantly localized to the plastid and cytosol, suggesting a possible redistribution of PSS1 during infection, potentially to facilitate nonhost immunity. PSS1 is also associated with thioglucoside glucohydrolase 1 (TGG1), a key enzyme in glucosinolate (GLS) metabolism, and to coproporphyrinogen III oxidase (LIN2), involved in tetrapyrrole biosynthesis and reactive oxygen species (ROS) production linked to defense-related cell death.

**Conclusions:**

We hypothesize that PSS1 contributes to a positive feedback loop that enhances multiple defense pathways. Based on consistent enrichment and functional relevance, we nominate TGG1, LIN2, and H(^+^)-ATPase 6 as priority candidates for functional validation in the context of PSS1-mediated nonhost immunity.

## Introduction

Proteins rarely function in isolation; instead, they carry out biological processes through dynamic interactions with other proteins, governed by chemical forces such as hydrogen bonding, salt bridges, and Hydrophobic interactions (Gingras et al., 2007; Lalonde et al., 2008; Syafrizayanti et al., 2014) Elucidating these protein–protein interactions (PPIs) is crucial for understanding the biochemical functions of novel proteins, particularly in response to environmental or biological stimuli. Mapping these interactions enables the reconstruction of dynamic and complex signaling networks (Morsy et al., 2008; Struk et al., 2019; Revathi Paramasivam et al., 2021).

Conventional methods for studying PPIs, such as biochemical fractionation and tandem affinity purification (TAP) followed by mass spectrometry (MS), have advanced our knowledge of stable, high-affinity protein complexes (Kim et al., 2016; Gingras et al., 2007; Oeffinger, 2012; Bontinck et al., 2018). However, these approaches often fail to detect weak, transient, or spatially restricted interactions critical for stress and immune responses (Oeffinger, 2012; Bontinck et al., 2018). To overcome these limitations, proximity labeling (PL) techniques have emerged as powerful alternatives. These methods use engineered biotin ligases fused to a bait protein to biotinylate nearby proteins within nanometer-scale proximity, enabling their enrichment and identification by MS (Kim and Roux, 2016; Mair et al., 2019; Yang et al., 2021).

BioID and its derivatives (BioID2, TurboID, miniTurbo) are widely used for tagging proximal proteins in living cells without crosslinking agents or toxic cofactors (Branon et al., 2018; Mair and Bergmann, 2022; Guo et al., 2023; Suzuki et al., 2023). TurboID and miniTurbo, derived from *E. coliBirA*, offer rapid biotinylation at physiological temperatures and are well-suited for plant systems. Importantly, miniTurbo was selected for this study due to its reduced background activity compared to TurboID, minimizing nonspecific labeling in plant tissues (Branon et al., 2018; Mair and Bergmann, 2022). Unlike APEX-based systems, which require hydrogen peroxide and may introduce oxidative stress, TurboID systems function under mild conditions and have been successfully used to map interactomes in diverse plant tissues and organelles (Nguyen et al., 2020; Dumrongprechachan et al., 2021; Yang et al., 2021).

The miniTurbo enzyme is particularly attractive for plant applications because of its lower background activity compared to TurboID, which minimizes nonspecific labeling. Applications in *Arabidopsis*, *Nicotiana benthamiana*, and *Marchantia polymorpha* have shown that miniTurbo-tagged bait proteins can label their interactors in the cytoplasm, nucleus, and plasma membrane with high fidelity (Branon et al., 2018; Mair and Bergmann, 2022; Melkonian et al., 2022, Guo et al., 2023). Moreover, this approach has proven useful in identifying host targets of pathogen effectors and key regulators of plant immune responses (Zhang Y. et al., 2019; Shi et al., 2023).

The Arabidopsis *PSS1* gene encodes a glycine-rich integral plasma membrane protein implicated in nonhost immunity against *Phytophthora sojae* and *Fusarium virguliforme* (Sumit et al., 2012; Wang et al., 2018). While transgenic soybean lines overexpressing *PSS1* exhibit enhanced resistance, the molecular basis of PSS1-mediated nonhost immunity mechanisms remains unclear. We hypothesize that PSS1 regulates defense-responsive enzymes whose proximity is dynamically enhanced in response to *P. sojae* infection. To test this hypothesis, we applied miniTurbo-based proximity labeling to uncover candidate interactors of PSS1. We generated stable *Arabidopsis* transgenic lines expressing miniTurbo fused to either the wild-type PSS1 or the PSS1^G119D^ mutant protein which is defective in nonhost immunity function. Plants were infected with *P. sojae* or treated with water (mock) in the presence of biotin, and biotinylated proteins were enriched and identified via mass spectrometry. Comparative analysis revealed proteins specifically enriched in the wild-type *PSS1* background but not in the PSS1^G119D^-background following *P. sojae* infection. These findings suggest that *PSS1* may coordinate multiple defense mechanisms to confer broad-spectrum nonhost immunity in Arabidopsis. This work laid the foundation for understanding the protein network through which PSS1 contributes to the broad-spectrum nonhost immunity.

## Experimental procedures

### Plant materials used in this study

*Arabidopsis thaliana* ecotype Col-0 was used as a wild-type control across all experiments. The null mutant *pss1-2* (SALK_090245C) was included in selected experiments to assess the functional relevance of *PSS1*-dependent associated protein network (Wang et al., 2018). In our earlier study the homozygosity of the *pss1–2* mutant was established through molecular analysis (Wang et al., 2018). The *miniTurbo* transgenic lines *(PSS1*- *V5-miniTurbo*, *PSS1^G119D^-V5-miniTurbo*, *miniTurbo-V5-PSS1*, and *miniTurbo-V5-PSS1^G119D^*) were included in specific subsets depending on the experimental objective.

Seeds were surface-sterilized in 70% ethanol followed by 33% (v/v) bleach, then rinsed several times with sterile double-distilled water before sowing on agar Petri plates. Seedlings were grown for 10 days on half-strength solid MS medium (Sigma, USA) containing 1% plant agar (Plant Media, USA) under a 16 h light/8 h dark cycle at 22°C. Soil-grown Arabidopsis plants were maintained under the same photoperiod and temperature conditions, watered regularly, and fertilized weekly (Clough and Bent, 1998).

### Cloning of miniTurbo fusion constructs

A 1.3 kb PSS1 promoter and 1.2 kb 3′-end fragment were PCR-amplified from *Arabidopsis thaliana* Col-0 genomic DNA using Q5 polymerase (NEB). Primers incorporated restriction sites (*Xba*I, *Nco*I, *Asc*I, *Avr*II, *Sma*I) for downstream cloning. The fragments were fused via overlapping PCR and inserted into *pBluescript KS II* (Alting-Mees and Short, 1989) then subcloned into the binary vector *pTF101.1* to generate *pTF-PSS1Prom-3′.*

For N-terminal fusions, miniTurbo-V5 and wildtype or mutant PSS1 ORFs (G119D) were PCR-amplified and fused via overlapping PCR. Products were cloned into *pBluescript KS II* (Alting-Mees and Short, 1989) and inserted into *pTF-PSS1Prom-3′* to yield *pTF-PSS1Prom::miniTurbo-V5-PSS1-3′* and *pTF-PSS1Prom::miniTurbo-V5-PSS1^G119D^-3′*.

For C-terminal fusions, V5 and miniTurbo fragments were fused to form V5-miniTurbo. Wildtype and mutant PSS1 ORFs were fused to this cassette via overlapping PCR, cloned into *pBluescript KS II* (Alting-Mees and Short, 1989), and inserted into *pTF-PSS1Prom-3′* to generate *pTF-PSS1Prom::PSS1-V5-miniTurbo-3′* and *pTF-PSS1Prom::PSS1^G119D^-V5-miniTurbo-3′*. Construct maps and validation data are shown in **Supplementary Figure 1**; plasmid details are listed in **Supplementary Table 2**. The details of cloning methodologies can be found in **Supplementary Methods**.

### Development of miniTurbo transgenic plants and complementation analysis

*Arabidopsis thaliana* transformation was carried out using individual *Agrobacterium tumefaciens* EHA101 strains harboring one of the four miniTurbo fusion constructs under the control of the native *PSS1* promoter and 3′ regulatory regions. Bacterial cultures were resuspended in infiltration buffer and adjusted to an OD_600_ of 0.5 prior to use. The T-DNA molecules containing each of the gene fusions, PSS1-V5-miniTurbo, PSS1^G119D^-V5-miniTurbo, miniTurbo-V5-PSS1, or miniTurbo-V5-PSS1^G119D^, were introduced into the *pss1–2* null mutant via the floral dip transformation method (Clough and Bent, 1998). At least three independent T_1_ lines were generated for each construct. The primers and plasmids generated in this study are listed in **Supplementary Tables 1**, **2**.

T_2_ plants were screened twice for glufosinate ammonium (60 µg/ml; Basta) resistance at a four-day interval. Ten T_2_ seedlings per line exhibiting ~75% survival rates were transplanted into soil. T_2:3_ lines showing 100% resistance were selected for further propagation. Transgenic plants were genotyped by PCR, and *PSS1^G119D^* lines were confirmed via digestion of PCR amplicons with the restriction enzyme *Aci*I (New England Biolabs, USA) to verify the G-to-A substitution responsible for the G119D mutation (**Supplementary Figure 1**). Expression of fusion proteins was validated by western blot using an anti-V5 antibody (Proteintech, USA), and functional biotin ligase activity was confirmed by detecting *cis*-biotinylated proteins using HRP-conjugated streptavidin (Abcam, USA) (**Supplementary Figure 2**). Homozygous T_4_ plants were used for all proximity labeling experiments.

For phenotypic assays, detached leaves from 21-day-old transgenic *Arabidopsis* seedlings expressing PSS1-V5-miniTurbo, PSS1^G119D^-V5-miniTurbo, miniTurbo-V5-PSS1, or miniTurbo-V5-PSS1^G119D^ were placed on moist Whatman filter paper and inoculated with *P. sojae* zoospores (10^6^ zoospores/mL) under high humidity conditions. Disease progression was imaged 72 h post-inoculation. Infected and dead cells were visualized by trypan blue staining by following established protocols (Sumit et al., 2012; Wang et al., 2018; Koch and Slusarenko, 1990; Kambakam et al., 2021). Across all experiments, PSS1^G119D^-V5-miniTurbo and miniTurbo-V5-PSS1^G119D^ consistently exhibited a susceptible phenotype, confirming the loss of function in the mutant background. In contrast, wild−type fusion constructs PSS1-V5-miniTurbo and miniTurbo-V5-PSS1, reproducibly restored a resistant phenotype in *pss1–2* background, demonstrating successful complementation.

### Protein isolation and immunoblotting

Total protein was extracted by homogenizing 500 mg of frozen, ground plant tissue in 1 mL chilled lysis buffer (50 mM Tris-HCl pH 7.5, 150 mM NaCl, 10% glycerol, 0.1% NP-40, 1 mM EDTA, 1 mM DTT, and protease inhibitor cocktail; Roche, USA) using a pre-cooled mortar and pestle. Homogenates were transferred to microcentrifuge tubes and centrifuged at 16,000 × g for 10 min at 4°C (Eppendorf, Germany). The supernatant was collected on ice, and protein concentration was determined using the Bradford assay.

For immunoblotting, protein extracts were mixed 1:1 with Laemmli sample buffer (3.0 mL H_2_O, 1.2 mL 1 M Tris-HCl pH 6.8, 2.4 mL glycerol, 0.48 g SDS, 60 µL 10% bromophenol blue, and 10% [v/v] β-mercaptoethanol), boiled at 95 °C for 10 min, and 20-30 µg of proteins were separated by SDS-PAGE. Proteins were transferred to nitrocellulose membranes (Bio-Rad, USA), and transfer efficiency was verified by Ponceau S staining (40% methanol, 15% glacial acetic acid, 0.25% Ponceau S). Rubisco large subunit staining was used to assess loading consistency.

Membranes were blocked with 2.5–5% (w/v) skim milk (Nestlé, USA) or BSA (Fisher, USA) in PBS-T or TBS-T, depending on the antibody. V5-tagged miniTurbo proteins were detected using a mouse monoclonal anti-V5 antibody (Proteintech, USA) overnight at 4°C, followed by HRP-conjugated anti-mouse IgG (Promega, USA) for 1 h at room temperature. Biotinylated proteins were detected using streptavidin-HRP (Abcam, USA) for 40–60 min at room temperature. Signals were visualized using Clarity Western ECL substrate (Bio-Rad, USA) and imaged with GEL DOC XR+ (Bio-Rad, USA).

### Sample preparation for LC-MS/MS analysis

Ten-day-old *Arabidopsis* seedlings expressing PSS1-V5-miniTurbo, PSS1^G119D^-V5-miniTurbo, miniTurbo-V5-PSS1, or miniTurbo-V5-PSS1^G119D^ were vertically grown on half-strength Murashige and Skoog (MS) medium and transferred into sterile six-well plates containing water for a 2-day acclimation period. On day 2, seedlings were inoculated with a *P. sojae* zoospore suspension (10^6^ zoospores/mL) and incubated in the dark. Mock controls were treated with sterile water. Four hours post-inoculation, biotin (Sigma, USA) was added to a final concentration of 250 µM, and seedlings were incubated for an additional 20 h in the dark at room temperature (RT). Following labeling, plants were rinsed three times with sterile deionized water (5 min each) and blotted dry on filter paper. This experiment was performed nine times, with seedlings from three independent experiments pooled for each biological replicate and total of three biological replicates. Pooling was used to reduce experimental variability and enhance protein identification depth by averaging biological noise across replicates.

Plant tissues were weighed, flash-frozen, and ground in liquid nitrogen using a chilled mortar and pestle. Total protein was extracted using RIPA buffer (50 mM Tris-HCl pH 7.5, 150 mM NaCl, 0.5% or 1% NP-40, 1% sodium deoxycholate, 0.1% SDS, and 1× protease inhibitor cocktail; Roche, USA). The lysates were centrifuged twice at 16,000 × *g* for 10 minutes at 4°C (Mair et al., 2019).

To remove excess free biotin, protein extracts were desalted using PD-10 columns (Sigma, USA) equilibrated with extraction buffer lacking protease inhibitors by following the manufacturer’s instructions. After desalting, 500 µL of 1× protease inhibitor cocktail was added to each sample. Protein concentrations were determined by Bradford assay.

**Figure 1:**
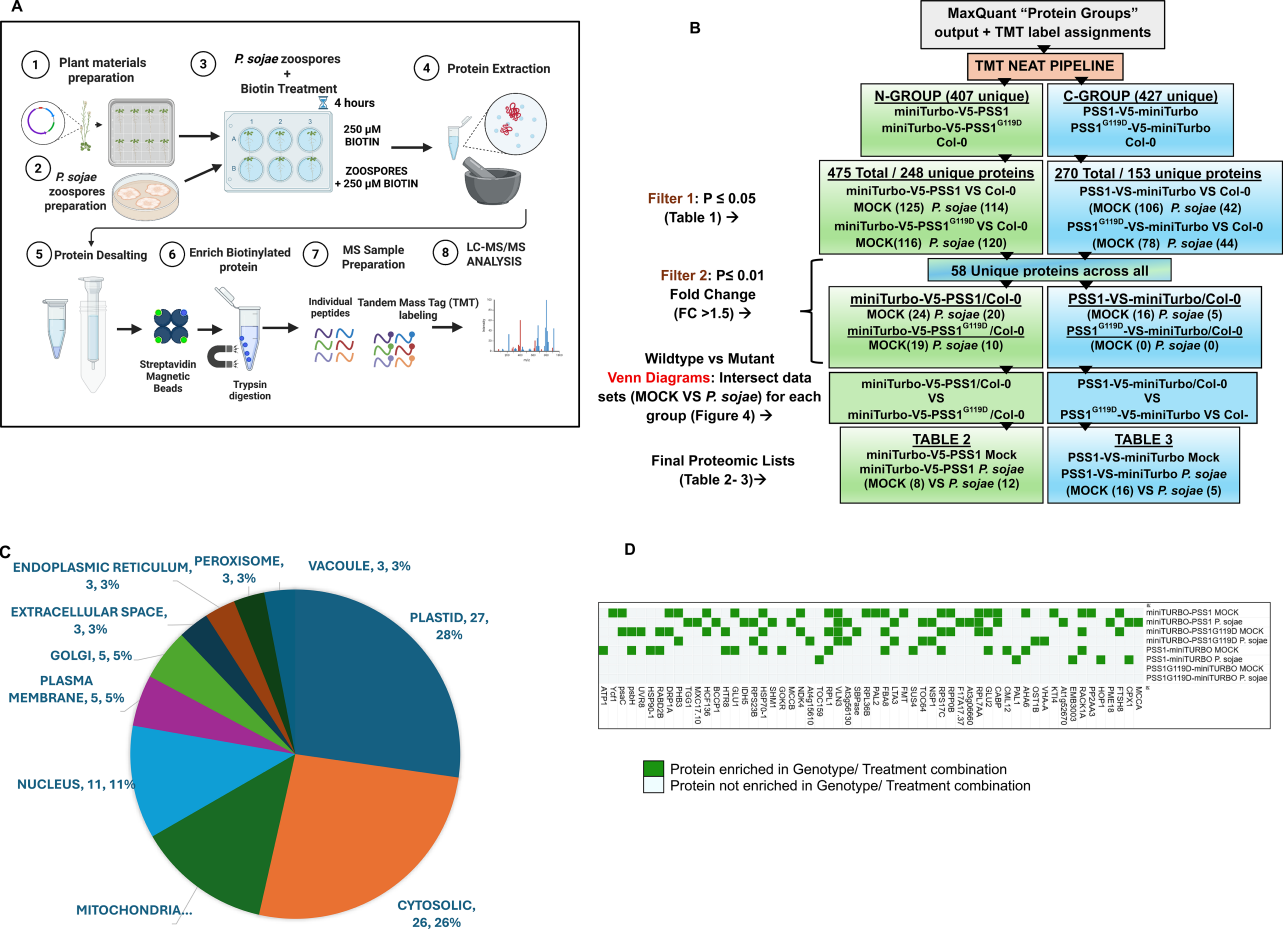
MiniTurbo proximity labeling workflow and subcellular distribution of PSS1-associated proteins identified by mass spectrometry. **(A)** Schematic overview of the experimental workflow for miniTurbo-based proximity labeling to identify PSS1-associated interactomes. Arabidopsis seedlings (Col-0, miniTurbo-V5-PSS1, and miniTurbo-V5-PSS1^G119D^) were grown on MS medium for 10 days and acclimatized in distilled water for 2 days. Twelve-day-old seedlings were inoculated with *P. sojae* zoospores for 4 hours, followed by treatment with 250 µM biotin for 20 hours in the dark at room temperature. Mock controls receive biotin under identical conditions without infection. Each sample consisted of pooled tissue from 20–30 seedlings. The experiment was performed nine times and pooled into three biological replicates. Tissues from 36 samples were homogenized in liquid nitrogen for protein extraction and desalting. Biotinylated proteins were enriched using streptavidin beads, digested with trypsin/LysC, labeled with TMT18pro, and analyzed by LC-MS/MS. Illustration created with BioRender (www.BioRender.com). **(B)** Schematic overview of the mass spectrometry data analysis workflow. Raw data were processed using MaxQuant and the TMT-NEAT pipeline. Proteins with normalized abundance ≤ 0.05 relative to Col-0 were selected, yielding 58 unique proteins across genotypes. Secondly, proteins were considered significantly enriched if they met both a fold change > 1.5 and p-value < 0.01 when comparing transgenic lines expressing N- or C-terminal miniTurbo-V5 fusion constructs to Col-0 controls. **(C)** Pie chart summarizing the predicted subcellular localization of the 58 enriched proteins (fold change > 1.5; p < 0.01), based on annotations from the SUBA5 database (Hooper et al., 2022). Localization categories include plastids (27%), cytosol (26%), mitochondria (13%), nucleus (11%), plasma membrane (5%), Golgi apparatus (5%), extracellular space (3%), endoplasmic reticulum (3%), peroxisome (3%), and vacuole (3%). Proteins with multiple predicted localizations were included in all relevant categories. **(D)** Binary heatmap showing the enrichment patterns of the 58 Arabidopsis proteins across genotypes and treatment conditions. Columns represent individual proteins; rows represent genotype-treatment combinations. Each cell indicates whether a given protein was enriched under the corresponding condition. Heatmap was generated using Morpheus.

Biotinylated proteins were enriched using Dynabeads MyOne Streptavidin C1 beads (Thermo Fisher Scientific, USA). For each 6.5 mg of desalted protein, 200 µL of bead slurry was used. Beads were pre-washed and equilibrated in binding buffer, then incubated with protein extracts overnight (15 h) at 4°C on a rotisserie rotator to ensure uniform binding. The next day, the beads were separated using a magnetic rack and subjected to a stringent wash protocol in the following order: once with cold extraction buffer (1 mL, 4°C), once with 1 M KCl, once with 100 mM Na_2_CO_3_, once with 2 M urea in 10 mM Tris-HCl (pH 7.5), and six washes with 50 mM Tris-HCl (pH 7.5). Finally, beads were resuspended in 200 µL of 50 mM Tris-HCl (pH 7.5) (**Figure 1A**).

To verify enrichment of biotinylated proteins, 5% of the bead slurry was boiled at 95°C for 10 minutes in 50 µL of 2× SDS-PAGE loading buffer containing 20 mM DTT and 2 mM biotin. The eluate was analyzed by western blot. The remaining beads for downstream on bead digestion protocol were flash-frozen in liquid nitrogen and stored at –80°C until LC-MS/MS analysis (Mair et al., 2019).

### Mass spectrometry analysis

Enriched proteins were eluted from streptavidin beads by incubating at 95°C for 10 minutes in 1× S-Trap lysis buffer (5% SDS, 50 mM TEAB, pH 8.5) supplemented with 12.5 mM biotin. Eluates were processed using S-Trap micro columns (ProtiFi, USA; Cat# C02-micro-80) following the manufacturer’s protocol. Protein samples were reduced by adding 2 mM TCEP and alkylated with 50 mM iodoacetamide (IAA), then digested overnight at 37°C using 100 ng each of trypsin and Lys-C. Resulting peptides were desalted using Sep-Pak C18 columns (Waters, USA) (Song et al., 2020). Peptides were labeled using Tandem Mass Tag (TMTpro 18-plex) reagents (Thermo Fisher Scientific, USA) as previously described (Song et al., 2020). TMTpro 18-plex label assignments for each sample are provided in **Supplementary Table 4**.

Labeling reactions were quenched with 5% hydroxylamine and pooled. Pooled samples were fractionated using the Pierce High pH Reversed-Phase Peptide Fractionation Kit (Thermo Fisher Scientific) per manufacturer instructions. The resulting eight fractions were concatenated to four pooled sets (1 + 5, 2 + 6, 3 + 7, 4 + 8), dried using a SpeedVac, and reconstituted in 0.1% TFA prepared with Optima™-grade H_2_O (Fisher Scientific).

For LC-MS/MS analysis, 1.5 µg of peptides from each sample (20 µL injection volume) were analyzed using liquid chromatography coupled to high-field asymmetric waveform ion mobility spectrometry and tandem mass spectrometry (LC-FAIMS-MS/MS). Samples were separated on a Vanquish Neo UHPLC system coupled to an Orbitrap Exploris 480 mass spectrometer using a μPAC Neo 110 cm column (PharmaFluidics). Flow was maintained at 300 nL/min. The EASY-Spray source was connected to a FAIMS Duo Pro interface, operating with compensation voltages of –45 V and –65 V (Clark et al., 2021).

Raw MS/MS spectra were searched against the *Arabidopsis thaliana* TAIR10 proteome (release: June 2011; downloaded from Arabidopsis.org, March 2024) and the *Phytophthora sojae* proteome (downloaded from NCBI, March 2024), supplemented with a reverse decoy database, common contaminants, and the custom PSS1-V5-miniTurbo sequence. Searches were performed using the Andromeda search engine within MaxQuant (v1.6.7.0) (Tyanova et al., 2016). Trypsin/Lys-C specificity was set, allowing up to two missed cleavages. Carbamidomethylation of cysteine was specified as a fixed modification, while methionine oxidation and protein N-terminal acetylation were treated as variable modifications. A minimum peptide length of seven amino acids was required, and identifications were filtered at a 1% false discovery rate at both peptide-spectrum match and protein levels using MaxQuant’s built-in target-decoy approach.

### Statistical analyses

Quantitative and statistical analyses were conducted using the TMT-NEAT Analysis pipeline (Clark et al., 2021). MaxQuant “ProteinGroups” output and a metadata file specifying TMT label assignments were provided as input (**Figure 1**). Initial processing included trimming and relabeling, and the removal of known contaminants. Data normalization was performed using both sample loading and internal reference normalization to ensure comparability across TMT runs.

Quality control included visual inspection of boxplots and principal component analysis (PCA) (**Supplementary Figure 5**). PCA was performed and visualized using the ggbiplot R package as part of the TMT-NEAT analysis pipeline. Volcano plots were generated using VolcaNoseR (https://goedhart.shinyapps.io/VolcaNoseR/) (Goedhart and Luijsterburg, 2020).

**Figure 2:**
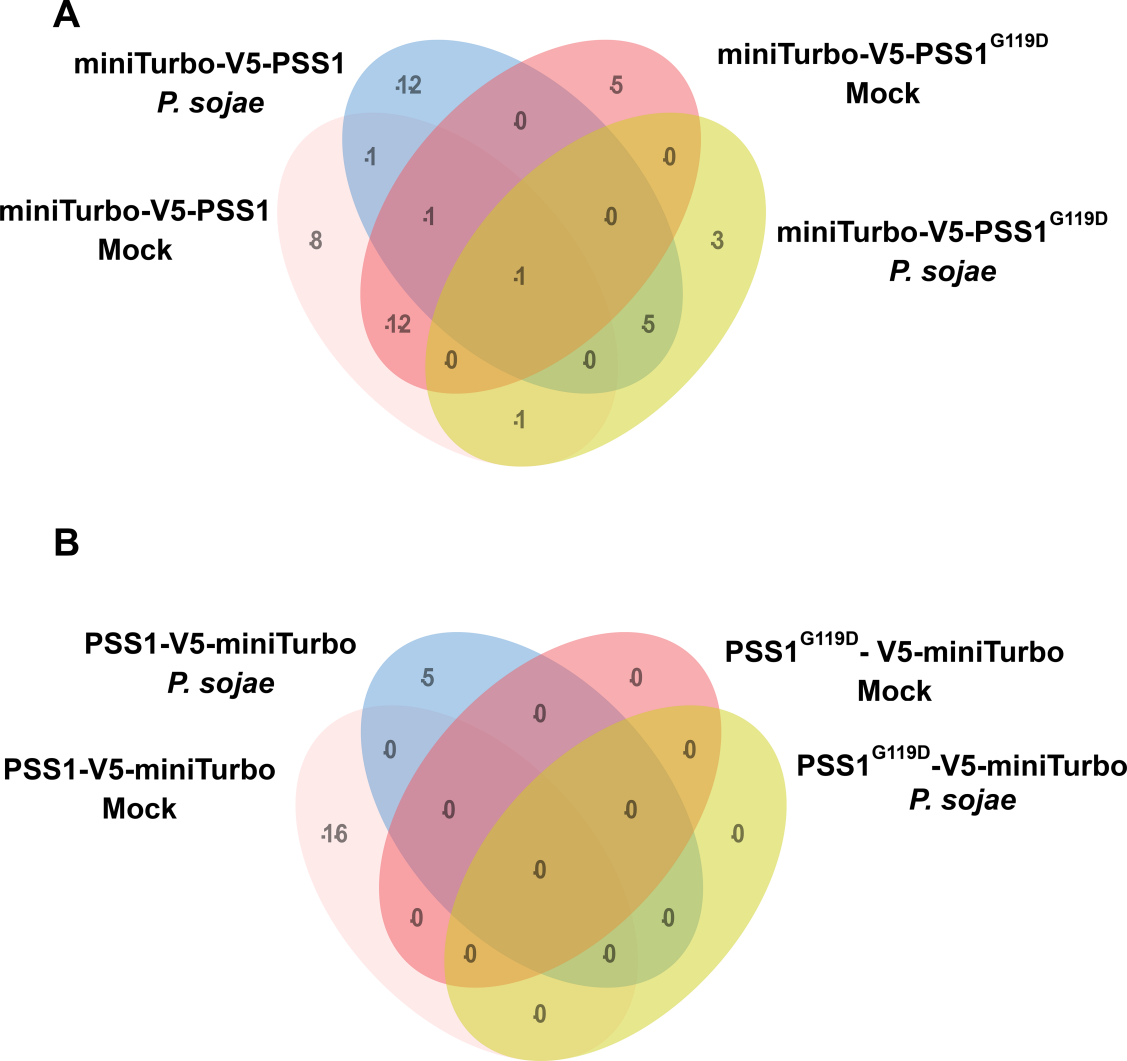
Classification of enriched proteins identified by miniTurbo proximity labeling. **(A)** Venn diagram showing the distribution of 49 proteins enriched in miniTurbo-V5-PSS1 and miniTurbo-V5-PSS1^G119D^ lines under mock and *P. sojae* infection conditions. Of these, 8 proteins were uniquely enriched under mock treatment, 12 under *P. sojae* infection, and 1 protein was shared between both conditions. **(B)** Venn diagram showing the distribution of 21 proteins enriched in PSS1-V5-miniTurbo and PSS1^G119D^-V5-miniTurbo lines under mock and *P. sojae* infection conditions. Sixteen proteins were uniquely enriched under mock treatment, 5 under *P. sojae* infection, with no overlap between conditions.

**Figure 3:**
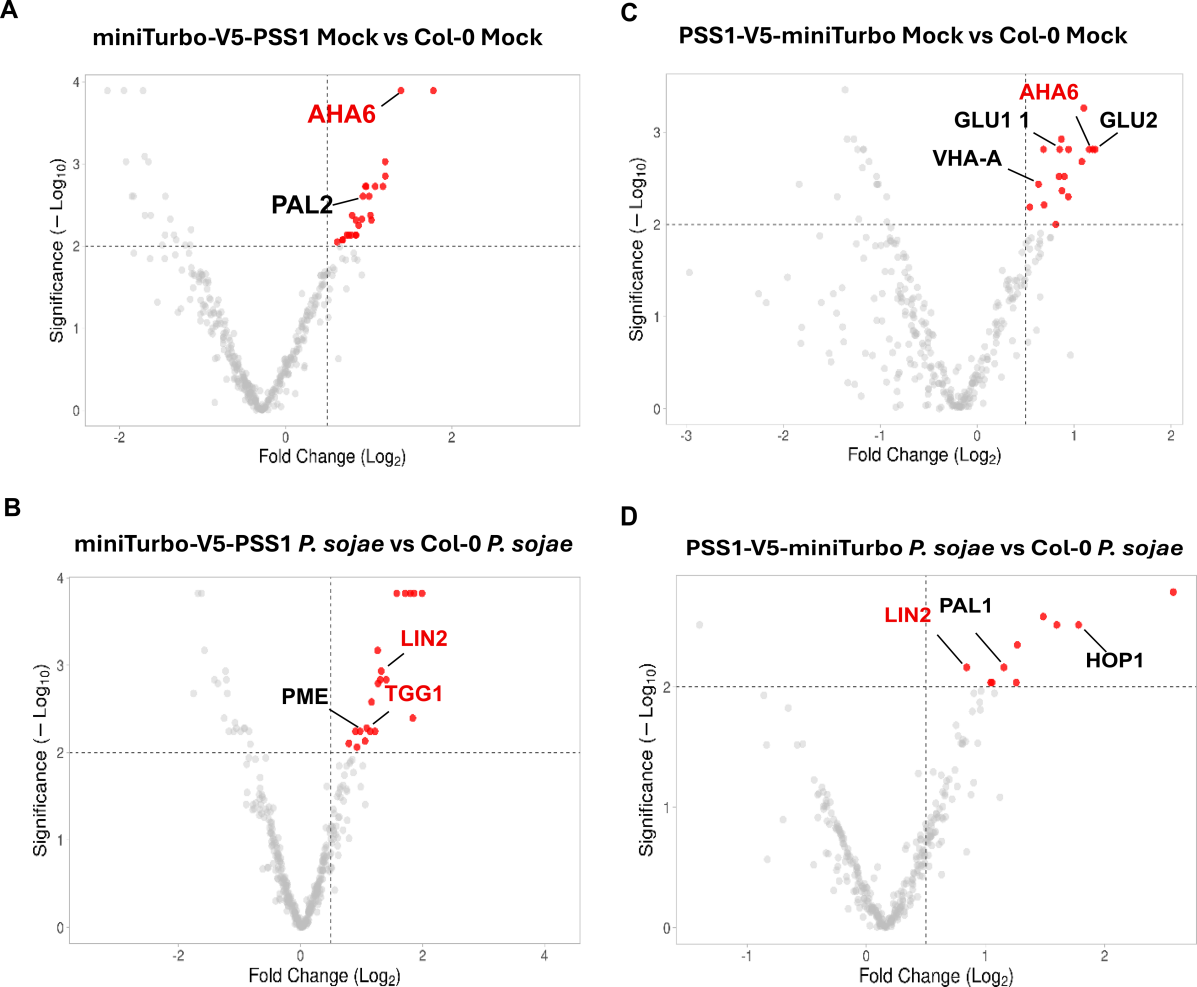
Volcano plots show significantly enriched proteins in miniTurbo-V5-PSS1 and PSS1-V5-miniTurbo Mock and *P. sojae* challenged samples. **(A)** Candidates enriched in miniTurbo-V5-PSS1 Mock. **(B)** Candidates enriched in miniTurbo-V5-PSS1 *P. sojae***(C)** Candidates enriched in PSS1-V5-miniTurbo Mock. **(D)** Candidates enriched in PSS1-V5-miniTurbo *P. sojae*. Candidates were filtered using stringent cutoffs log_2_FC > 0.5 and log_2_ pvalue > 2. Plots were generated using VolcaNoseR. Enriched proteins are labeled.

To account for multiple hypothesis testing, *p*-values were adjusted using the Benjamin-Hochberg (BH) procedure to control the False discovery rate (FDR). For initial candidate identification (**Table 1**), proteins with BH-adjusted p-values ≤ 0.05 were considered enriched over Col-0 nontransgenic controls. For downstream analyses (**Tables 2**, **3**; **Figures 2**, **3**), a more stringent threshold of p ≤ 0.01 and fold change > 1.5 was applied to define robust enrichment.

**Table 1:** Summary of proteomic data set identified by mass spectrometry.

N-Fusion group				C-Fusion group			
475 proteins				270 proteins			
miniTurbo-V5-PSS1 Mock	miniTurbo-V5-PSS1 *P. sojae*	miniTurbo-V5-PSS1^G119D^ Mock	miniTurbo-V5-PSS1^G119D^*P. sojae*	PSS1-V5-miniTurbo Mock	PSS1-V5-miniTurbo Mock *P. sojae*	PSS1^G119D^-V5-miniTurbo Mock	PSS1^G119D^-V5-miniTurbo *P. sojae*
125	114	116	120	106	42	78	44

Summary of differentially enriched proteins (p ≤ 0.05) identified in transgenic Arabidopsis lines expressing N-terminal (N-Fusion) or C-terminal (C-Fusion) miniTurbo-tagged PSS1 variants, relative to nontransgenic Col-0 controls. A total of 475 proteins were enriched in the N-Fusion group and 270 proteins in the C-Fusion group across mock and *P. sojae* treatments. The number of significantly enriched proteins for each genotype-treatment combination is indicated. Complete mass spectrometry data are provided in Supplemental Datasets 1 and 2.

**Table 2:** *Arabidopsis* proteins uniquely enriched in miniTurbo-V5-PSS1 transgenic lines under mock-treated and P. sojae-infected conditions.

miniTurbo-V5-PSS1 Mock									
Gene ID	Protein	Abbreviation	FC	Log2 FC	*p*-value	Log10 (*p*-value)	Location	Function summary	Reference
AT3G52140.3	Tetratricopeptide repeat (TPR)-containing protein	TPR	3.43	1.779	0.00	3.892	nucleus	Protein-protein interactions, scaffolding and cellular transport	Rosado et al., 2006
AT2G07560.1	H(+)-atpase 6	AHA6	2.62	1.39	0.00	3.892	plasma membrane	Modulates signal transduction, development, and stress responses	Elmore and Coaker, 2011; Michalak et al, 2022
AT2G41220.1	Glutamate synthase 2	GLU2/GS2	2.03	1.199	0.00	3.027	plastid	Connects nitrogen assimilation to defense signaling	Ding et al., 2023
AT3G53260.1	Phenylalanine ammonia-lyase 2	PAL2	2.01	1.022	0.00	2.374	peroxisome	Component of ribosome; involved in RNA binding, ribosome assembly, and possibly stress signaling	Huang et al., 2010; Mauch-Mani and Slusarenko, 1996
AT5G04140.1	Glutamate synthase 1	GLU1	1.91	1.006	0.00	2.606	plastid	Glutamate synthesizes	Seifi et al., 2013
AT1G13320.2	Protein phosphatase 2A subunit A3	PP2AA3	1.74	0.931	0.00	2.606	cytosol	Negative regulator of Plant immunity	Segonzac et al., 2014
AT1G73260.1	Kunitz trypsin inhibitor 1	KTI1	1.61	0.802	0.01	2.374	extracellular	Modulates Program cell death in plant pathogen interactions	Li et al., 2008
AT3G53740.1	Ribosomal protein L36e family protein	RPL36e	1.54	0.686	0.01	2.075	cytosol	Acts as a transcription factor to modulate immunity related gene expression	Fakih et al., 2023
ATCG01130.1	Ycf1 protein	YCF1	3.43	0.623	0.00	2.049	plastid	Manages cellular stress and chloroplast gene expression	Wang et al., 2025

miniTurbo-V5-PSS1 *P. sojae*									
Gene ID	Protein	Abbreviation	FC	Log2 FC	p-value	Log10 (p-value)	Location	Function summary	Reference
AT5G16390.2	Chloroplastic acetyl-CoA carboxylase 1	CAC1	3.97	1.993744	0.01	2.132626	plastid	Regulates fatty acid biosynthesis which influences defense hormones	Zhao et al., 2022
AT3G06660.1	PAPA-1-like/Zinc finger (HIT type) protein	ZFP-HIT	3.63	1.862797	0.00	2.93352	nucleus	Transcriptional regulator; stress and development	Noman et al., 2019
AT5G24710.1	Transducin/WD40 repeat-like superfamily protein	WD40/ARC1	3.29	1.718926	0.01	2.104735	nucleus, plasma membrane	Scaffold for protein interactions; regulates cell cycle and RNA processing	Bashline et al., 2015
AT1G03475.1	Coproporphyrinogen III oxidase	LIN2/CPOX	2.51	1.32536	0.00	3.822822	plastid	Tetrapyrrole biosynthesis for chlorophyll and heme. Can activate plant immunity and disease resistance	Guo et al., 2013; Ishikawa et al., 2001
AT3G08030.2	DUF642 protein	DUF642	2.41	1.266	0.00	2.834	cytosol	Regulates pectin methylesterase activity; cell wall function	Xie and Wang, 2016; Cruz-Valderrama et al., 2019
AT1G03090.2	3-methylcrotonyl-CoA carboxylase 1	MCCA	2.33	1.222	0.00	3.823	mitochondrion	Catalyzes leucine degradation in mitochondria	Che et al., 2002
AT4G37930.1	Serine transhydroxymethyltransferase 1	SHMT1	2.25	1.165	0.01	2.243	mitochondrion	Helps reduce the accumulation of ROS which causes cell damage	Moreno et al., 2005
AT4G34030.1	3-methylcrotonyl-CoA carboxylase	MCCB	2.20	1.142	0.01	2.243	mitochondrion	Catalyzes a step in leucine breakdown in mitochondria	Che et al., 2002
AT5G26000.2	Thioglucoside glucohydrolase 1	TGG1	2.13	1.085	0.01	2.063	vacuole	Myrosinase enzyme; hydrolyzes glucosinolates for defense	Barth and Jander, 2006; Calmes et al., 2015; Tierens et al., 2001; Zhang K. et al., 2019; Pastorczyk and Bednarek, 2016; Bednarek et al., 2009
AT1G52670.1	Single hybrid motif superfamily protein	SHM	2.08	1.059	0.00	3.823	plastid	May mediate coenzyme metabolism and protein interactions	Chen et al., 2009
AT1G11580.1	Methylesterase PCR A	PME	1.97	0.977	0.00	3.823	extracellular	Pectin demethylesterification; cell wall remodeling	Lionetti et al., 2012
AT5G03290.1	Isocitrate dehydrogenase V	IDH-V	1.73	0.793	0.01	2.243	mitochondrion	Generate NADPH during oxidative stress	Leterrier et al., 2012

Proteins were identified based on significant enrichment (fold change > 1.5; p ≤ 0.01) relative to the corresponding nontransgenic Col-0 controls (e.g., transgenic mock *vs*. Col-0 mock).

**Table 3:** Arabidopsis proteins uniquely enriched in PSS1-V5-miniTurbo transgenic lines under mock-treated and P. sojae-infected conditions.

PSS1-V5 miniTurbo mock									
Gene ID	Protein	Abbreviation	FC	Log2 FC	p-value	Log10 (p-value)	Location SUBAcon	Function summary	Reference
AT2G41220.1	Glutamate synthase 2	GLU2/GS2	2.33	1.216	0.00	2.814	plastid	Connects nitrogen assimilation to defense signaling	Ding et al., 2023
AT2G07560.1	H(+)-atpase 6	H(+)-ATPase 6	2.28	1.193	0.00	2.814	plasma membrane	Modulates signal transduction, development, and stress responses	Elmore and Coaker, 2011; Michalak et al, 2022
AT5G02500.1	Heat shock cognate protein 70-1	HSC70-1	2.23	1.156	0.00	2.814	cytosol	Chaperone, ROS regulation	Berka et al., 2022
AT1G18080.1	Receptor for activated Kinase 1A	RACK1A	2.14	1.099	0.00	3.264	cytosol	Scaffold for protein interactions and signaling pathway regulator	Cheng et al., 2015
AT3G48420.1	Haloacid dehalogenase-like hydrolase (HAD) superfamily protein	HAD hydrolase	2.11	1.079	0.00	2.682	plastid	Drought tolerance and oxidative stress related hydrolase	Lee et al., 2022; Zan et al., 2023
AT3G10610.1	Ribosomal S17 family protein	RPS17C	1.92	0.939	0.01	2.301	cytosol	Protein synthesis and Plant stress responses	Barakat et al., 2001
ATCG00710.1	Photosystem II reaction center protein H	PSBH	1.92	0.94	0.00	2.815	plastid	Photosynthesis	Zhang et al., 2016
AT2G38700.1	Mevalonate diphosphate decarboxylase 1	MVD1	1.87	0.901	0.00	2.523	cytosol	Isoprenoid biosynthesis enzyme	Cho et al., 2022
AT4G35120.1	Galactose oxidase/kelch repeat superfamily protein;	Kelch repeat	1.84	0.875	0.00	2.366	cytosol	Regulate Phenylpropanoid biosynthesis by modulating PAL turnover	Zhang et al., 2014
AT5G47200.1	RAB gtpase homolog 1A	RAB1A	1.83	0.868	0.00	2.925	Golgi, endoplasmic reticulum	Regulate endomembrane trafficking	Tripathy et al., 2021

PSS1-V5 miniTurbo *P.sojae*									
Gene ID	Protein	Abbreviation	FC	Log2 FC	0.00	Log10 (p-value)	Location SUBAcon	Function summary	Reference
AT1G34430.1	2-oxoacid dehydrogenases acyltransferase family protein;	LTA1	5.98	2.577	0.01	2.789	plastid	E2 component of pyruvate dehydrogenase complex; involved in acyl group transfer and amino acid degradation	Lin et al., 2003
AT1G12270.1	stress-inducible protein, putative;	HOP1	3.43	1.782	0.01	2.516	nucleus	Putative regulator of abiotic stress responses; often upregulated under salt, drought, or pathogen stress	Fellerer et al., 2011
AT4G02510.1	translocon at the outer envelope membrane of chloroplasts 159	TOC159	3.03	1.600	0.00	2.516	plastid	GTPase receptor for chloroplast protein import; regulates biogenesis and is modulated by phosphorylation	Arronsson and Jarvis, 2011
AT2G37040.1	PHE ammonia lyase 1	PAL1	2.81	1.486	0.00	2.585	peroxisome	Converts phenylalanine to cinnamic acid; key enzyme in phenylpropanoid biosynthesis and defense	Huang et al., 2010; Mauch-Mani and Slusarenko, 1996
AT1G03475.1	Coproporphyrinogen III oxidase.	LIN2	2.41	1.268	0.01	2.350	plastid	Catalyzes a key step in tetrapyrrole biosynthesis for chlorophyll and heme; mutants show defense-related phenotypes. responses, usually activated by pathogen infection	Tanaka and Tanaka, 2007; Guo et al., 2013; Madsen et al., 1993

Proteins were identified based on significant enrichment (fold change > 1.5; p ≤ 0.01) relative to the corresponding nontransgenic Col-0 controls (e.g., transgenic mock *vs*. Col-0 mock).

### In silico protein characterization

The identified proteins were annotated for subcellular localization and functional information using UniProt (released in 2023; Bateman et al., 2023), eBAR (Waese et al., 2017), and the SUBA5 database (released in 2021-03, version 15A76d4; assessed June 2025; Hooper et al., 2022). The protein lists were gathered from different genotypes with or without *P. sojae* infection and compared using jVenn program (Bardou et al., 2014).

## Results

### Identification of the PSS1- and PSS1^G119D^- interactomes

In this study, we included two controls: (i) Col-0 to monitor the background biotynilation levels of proteins to facilitate identification of the proteins that were specifically biotinylated by the miniTurbo fusion genes consistently across all three biological replications; (ii) miniTurbo fused *PSS1^G119D^* to facilitate identification of the candidate PSS1-interacting proteins. We hypothesized that PSS1^G119D^ fails to interact with proteins that are involved in nonhost immunity.

**Figure 4:**
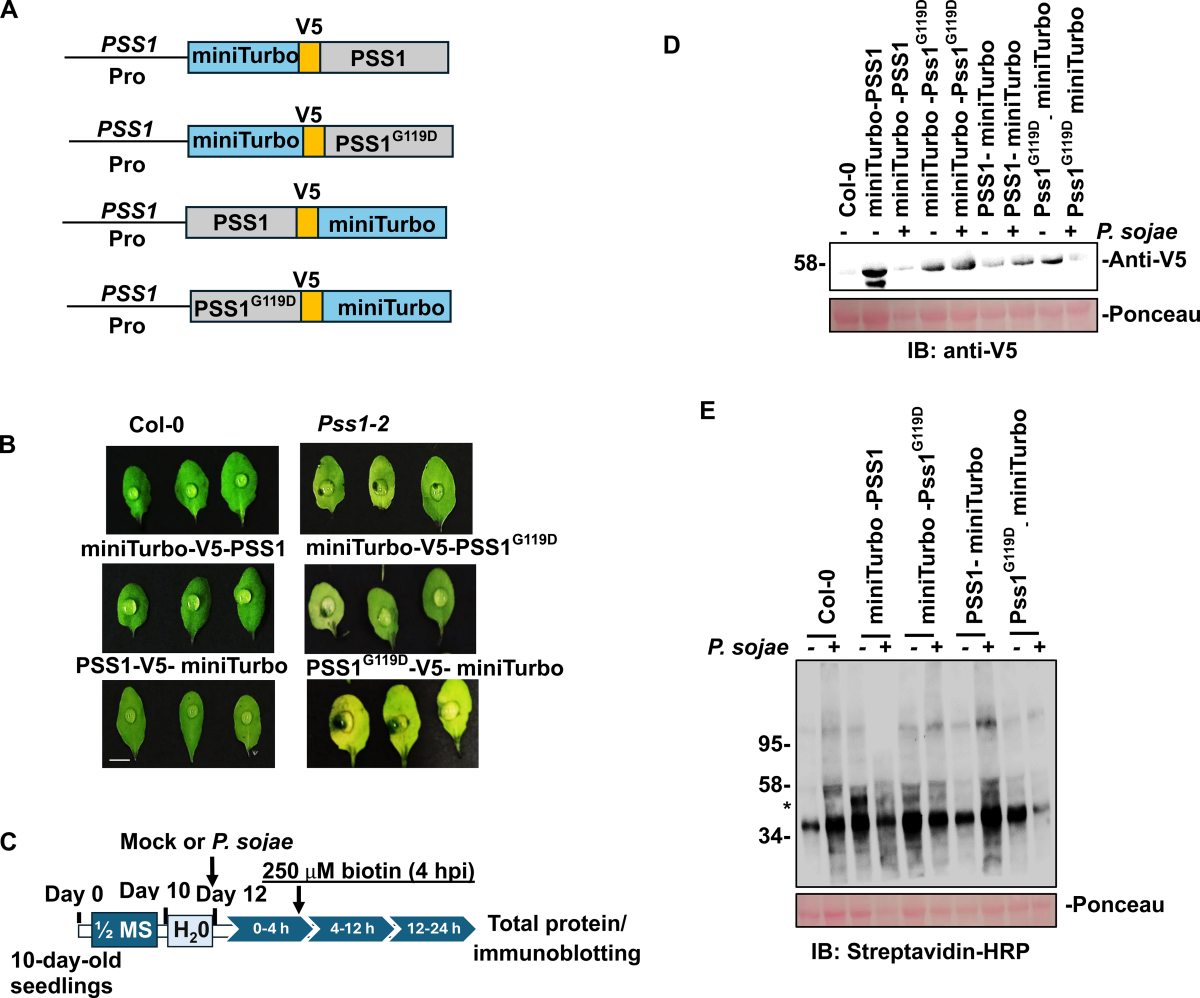
Experimental setup for miniTurbo proximity labeling in Arabidopsis. **(A)** Schematic representation of miniTurbo fusion gene constructs used for proximity labeling and mass spectrometry analysis. **(B)** Complementation of the nonhost resistance defect in the *pss1–2* mutant (SALK_090245C) by various miniTurbo fusion constructs. Detached leaves from 21-day-old Arabidopsis plants expressing miniTurbo-V5-PSS1, miniTurbo-V5-PSS1^G119D^, PSS1-V5-miniTurbo, or PSS1^G119D^-V5-miniTurbo, along with Col-0 and *pss1-2*, were inoculated with 10 µL of *P. sojae* zoospore suspension (10^6^ spores/mL). Scale bar = 5 mm. **(C)** Overview of the proximity labeling workflow. Arabidopsis seedlings were grown on ½ MS medium for 10 days and equilibrated in water for 2 days. Twelve-day-old seedlings of the indicated genotypes were inoculated with *P. sojae* zoospores for 4 hours, followed by treatment with 250 µM biotin and incubation for an additional 20 hours in the dark at room temperature. Each sample consisted of pooled tissue from 20–30 seedlings. Mock controls received biotin without *P. sojae* and were incubated under identical conditions. Tissues were homogenized for protein extraction and immunoblotting. **(D)** Immunoblot analysis of miniTurbo fusion protein expression. Twelve-day-old seedlings of Col-0, *pss1-2*, and transgenic lines were challenged with *P. sojae* zoospores (10^5^ spores/mL) or sterile water for 4 hours, followed by 250 µM biotin treatment for 20 hours. Protein extracts were probed with anti-V5 antibody. Ponceau staining served as a loading control. Molecular weights (kDa) are indicated. **(E)** Streptavidin-HRP blot detecting biotinylated proteins in total extracts from transgenic lines. Both N- and C-terminal miniTurbo fusions to PSS1 or PSS1^G119D^ were analyzed following mock or *P. sojae* treatment. Protein extracts were probed with Streptavidin-HRP. Cis-biotinylation is indicated by an asterisk (*). Ponceau staining served as a loading control. Molecular weights (kDa) are indicated.

Binary vector plasmids carrying *miniTurbo-V5-PSS1, miniTurbo-V5-PSS1^G119D^, PSS1-V5-miniTurbo*, and *PSS1^G119^-V5-miniTurbo* fusion genes were (**Figure 4A**, **Supplementary Figure 1A**) used to transform *A. thaliana*. To evaluate transgene expression, we first conducted transient expression assays in *N. benthamiana* via *Agrobacterium*-mediated infiltration. Leaf tissue was harvested 72 h post-infiltration for RNA extraction and RT-PCR for determining the transcription of all four fusion genes (**Supplementary Figure 1B**). Stable transgenic lines were generated in the null *pss1-2* (Salk_090245) mutant and homozygous lines were identified for each of the four fusion genes (**Supplementary Figures 1C, D**). Detached leaves from 21-day-old transgenic plants, wild-type Col-0, and *pss1–2* mutants were inoculated with *P. sojae* or treated with water (mock or control treatment). Complementation assays showed that only the expression of the wild-type *PSS1* fusion genes (*miniTurbo-V5-PSS1* and *PSS1-V5-miniTurbo*), but not the *PSS1^G119D^* variants (*miniTurbo-V5-PSS1^G119D^* and *PSS1^G119D^-V5-miniTurbo*), restored nonhost resistance (**Figure 4B**, **Supplementary Figure 2A**). The observed qualitative phenotypes were reproducible across independent transgenic lines (**Figure 4B**, **Supplementary Figure 2A**). Protein expression in transgenic lines was confirmed by immunoblotting using anti-V5 antibodies (**Supplementary Figure 2B**), and the enzymatic activity of the miniTurbo fusion proteins was verified by detecting biotinylated proteins in total extracts using streptavidin-HRP (**Supplementary Figure 2C**).

### The optimized biotin level and incubation period for trans-biotinylation of PSS1-associated proteins

The efficiency of the trans-biotinylation by the miniTurbo gene is influenced primarily by local biotin concentration and incubation duration, given that miniTurbo functions optimally at room temperature (Kim et al., 2016; Branon et al., 2018). To optimize trans-biotinylation efficiency for proximity labeling, we tested varying biotin concentrations and incubation periods using transgenic lines expressing fusion genes miniTurbo-V5-PSS1*, miniTurbo-V5-PSS1^G119D^, PSS1-V5-miniTurbo*, or *PSS1^G119D^-V5-miniTurbo*. Our goal was to optimize biotin labeling conditions that maximize the labeling efficiency while minimizing physiological disruption to the *Arabidopsis* and *P. sojae* interactions. Ten-day-old miniTurbo-V5-PSS1 seedlings were initially grown on vertical Petri plates containing half-strength MS medium, then transferred to sterile water in six-well plates for two days and subsequently incubated in biotin solutions. Biotin concentrations ranged from 0 to 250 µM for miniTurbo-V5-PSS1 and PSS1-V5-miniTurbo (**Supplementary Figures 3A, C**) and 0 to 500 µM miniTurbo-V5-PSS1^G119D^ and PSS1^G119D^ -V5-miniTurbo (**Supplementary Figures 3B, D**). To identify the most suitable conditions for efficient *in vivo cis*-biotinylation at room temperature, we also tested three incubation times, 3, 6, and 24 h, aiming to reduce the risk of false positives associated with prolonged exposure.

In miniTurbo-V5-PSS1 plants, increased biotinylated protein levels were detected after 3 and 6 h of incubation with biotin concentrations ranging from 25 to 250 µM. (**Supplementary Figure 3A**). This trend was not observed in the miniTurbo-V5-PSS1^G119D^ lines (**Supplementary Figure 3B**). After 24 h of biotin treatment, we observed robust *cis*- and *trans*-biotinylation in miniTurbo-V5-PSS1 lines with increasing biotin concentrations (0–250 µM), whereas wild-type Col-0 did not show such labeling even at 250 µM (**Supplementary Figure 3C**) In contrast, biotinylation levels in miniTurbo-V5-PSS1^G119D^ plants remained low across all tested concentrations (0–500 µM) for 24 h (**Supplementary Figure 3D**). Based on these observations, we selected 250 µM biotin and the 20-h incubation period as the optimal condition for profiling the PSS1- and PSS1^G119D^-interactomes.

We further assessed our selected 250 µM biotin for 20 h at room temperature labeling conditions. Transgenic and wild-type Col-0 seedlings were pre-treated in water for two days, then inoculated with *P. sojae* zoospore suspension (10^6^ spores/mL) for 4 h in the dark without agitation. Biotin was subsequently added to a final concentration of 250 µM, and plants were incubated for 20 h (**Figure 4C**). Protein extracts were analyzed via immunoblotting with anti-V5 antibody to confirm expression of the miniTurbo fusion proteins (**Figure 4D**). Probing with HRP-conjugated streptavidin detected a characteristic smear of biotinylated protein species in the *P. sojae*-infected leaf tissues from miniTurbo-V5-PSS1, miniTurbo-V5-PSS1^G119D^, PSS1-V5-miniTurbo, and PSS1^G119D^-V5-miniTurbo lines, validating successful labeling under the optimized conditions (250 µM biotin for 20 h) (**Figure 4E**).

### Proximity proteomes detected by miniTurbo-V5-PSS1, miniTurbo-V5-PSS1^G119D^, PSS1-V5-miniTurbo and PSS1^G119D^-V5-miniTurbo fusion genes

To identify candidate proteins that are associating with PSS1, we employed a miniTurbo-based proximity labeling strategy using four different fusion constructs: miniTurbo-V5-PSS1, miniTurbo-V5-PSS1^G119D^, PSS1-V5-miniTurbo, and PSS1^G119D^-V5-miniTurbo. 250 µM biotin was applied to transgenic *Arabidopsis* plants 4 h after inoculation with *P. sojae* zoospores and incubated for 20 h at room temperature in the dark (**Figure 1A**). Control samples were mock-treated and incubated with 250 µM biotin under similar conditions. We conducted the nine independent experiments. To reduce the experimental variability, the plant tissues of three random experiments from these nine were pooled to constitute one biological replicate. Total protein was extracted from plant tissues, and excess biotin was removed using desalting columns. Biotinylated proteins were enriched using streptavidin C1 beads. (**Figure 1A**, **Supplementary Figure 4A**). We also revalidated the transgenic lines by conducting dCAPS analysis (**Supplementary Figure 4B**). To confirm successful biotin labeling, 5% of the total bead volume was analyzed via immunoblotting using HRP-conjugated streptavidin (**Supplementary Figure 4C**). The remaining proteins were subjected to trypsin digestion and Tandem Mass Tag (TMT) labeling (**Figure 1A**; **Supplementary Table 3**), followed by LC-MS/MS analysis (**Supplementary Figure 2A**). Spectra were processed using MaxQuant, and differential protein enrichment was assessed using the TMT-NEAT pipeline (Clark et al., 2021).

From the LC-MS/MS data analyses (**Figure 1B**), we identified a total of 475 proteins (247 unique) in the miniTurbo-V5-PSS1/PSS1^G119D^ group and 270 proteins (153 unique) in the PSS1/PSS1^G119D^-V5-miniTurbo group that showed significant differential enrichment (p ≤ 0.05) relative to their respective Col-0 controls (**Table 1**; **Supplemental Data Sets**). Principal component analysis revealed clear separation between transgenic and non-transgenic Col-0 samples (**Supplementary Figure 5**).

To further classify the 475 and 270 Arabidopsis and *P. sojae* proteins, we quantified enrichment across mock and pathogen-challenged conditions. In the miniTurbo-V5-PSS1 *vs*. Col-0 comparison, 125 and 114 proteins were enriched in transgenic samples under mock and *P. sojae*-infected conditions, respectively (**Figure 1B**). In the miniTurbo-V5-PSS1^G119D^*vs*. Col-0 comparison, 116 and 120 proteins were enriched under mock and infected conditions, respectively. For the PSS1-V5-miniTurbo *vs*. Col-0 comparison, 106 and 42 proteins were enriched under mock and *P. sojae* -infected conditions, respectively. Similarly, in the miniTurbo-V5-PSS1^G119D^*vs*. Col-0 comparison, 78 and 44 proteins were enriched under mock and *P. sojae*-infected conditions (**Table 1**, **Figure 1B**).

Following the removal of 49 total/23 unique *P. sojae* proteins (Supplementary Data sets) from the 475 miniTurbo-V5-PSS1/PSS1^G119D^ group proteins and 65 total/36 unique *P. sojae* proteins (Supplementary Data sets) from the 270 PSS1/PSS1^G119D^-V5-miniTurbo group proteins, we applied a stringent enrichment threshold (p < 0.01 and fold change > 1.5) to prioritize 58 unique *Arabidopsis* proteins consistently enriched across all transgenic genotypes and treatment conditions (**Figures 1B-D**, **Supplementary Table 4**).

Subcellular localization analysis using the SUBA5 database (Hooper et al., 2022) predicted that 27% of these proteins were plastid-localized, 26% cytosolic, 13% mitochondrial, 11% nuclear, 5% associated with the plasma membrane, 5% Golgi-localized, and 3% each localized to the extracellular space, endoplasmic reticulum, peroxisome, or vacuole (**Figure 1C**; **Supplementary Table 4**).

Incorporation of the PSS1^G119D^ mutant as a control enabled us to distinguish proteins that interact specifically with the wild-type PSS1 protein and presumably involved in nonhost immunity from those that may associate nonspecifically in both PSS1 and mutant PSS1^G119D^ forms. To refine the list of candidate interactors, we performed a comparative analysis of the 58 prioritized proteins (**Supplementary Table 4**) using Venn diagrams (**Figures 2A, B**). A closer examination of enrichment patterns showed that the miniTurbo-V5-PSS1 group yielded eight proteins under mock conditions and 12 following *P. sojae* treatment (**Table 2**). In contrast, the PSS1-V5-miniTurbo group enriched 16 proteins under mock conditions and five upon *P. sojae* infection (**Table 3**).

In the *P. sojae*-infected miniTurbo-V5-PSS1 group, 12 proteins including chloroplastic acetylcoenzyme A carboxylase 1 (FC: 3.43, *p*-value: <0.01), PAPA-1-like family protein/zinc finger (HIT type) family protein (FC: 3.63, *p*-value: <0.01), Transducin/WD40 repeat-like superfamily protein (FC: 3.29, *p*-value: <0.01), Coproporphyrinogen III oxidase (LIN2) (FC: 2.51, *p*-value: <0.01), DUF642 protein with unknown function (FC: 2.41, *p*-value: <0.01), 3-methylcrotonyl-CoA carboxylase (FC: 2.33, *p*-value: <0.01), thioglucoside glucohydrolase 1 (TGG1) (FC: 2.13, *p*-value: 0.01), and 3-methylcrotonyl-CoA carboxylase subunits (MCCA) (FC: 2.33, *p*-value: <0.01) were uniquely enriched, suggesting that these are the potential PSS1-interacting proteins presumably involved in nonhost immunity (**Figures 2A**, **3**, **Table 2**).

Within the miniTurbo-V5-PSS1 mock group, we identified eight proteins including a tetratricopeptide repeat (TPR)-containing protein (FC: 3.43, *p*-value: <0.01), H^+^-ATPase (FC: 2.62, *p*-value: <0.01), protein phosphatase 2A subunit A3 (PP2AA3) (FC: 1.91, *p*-value: <0.01), phenylalanine ammonia-lyase 2 (PAL2 (FC: 2.03, *p*-value: <0.01)), YCF1 protein (FC: 1.54, *p*-value: <0.01), and glutamate synthase 1 (GLU 1) (FC: 2.01, *p*-value: <0.01) were enriched and can be considered as the candidate PSS1-interactors (**Figures 2A**, **3**, **Table 2**).

In the *P. sojae*-infected PSS1-V5-miniTurbo group, five proteins including 2-oxoacid dehydrogenases acyltransferase family protein (FC: 2.01, *p*-value: <0.01), stress-inducible protein, TOC159 (Translocon at the outer envelope membrane of chloroplasts 159) (FC: 3.03, *p*-value: <0.01), and Copropphyrinogen III oxidase (FC: 2.41, *p*-value: 0.01) were enriched and are most likely PSS1-interactors that could be involved in nonhost immunity (**Figures 2B**, **3**, **Table 3**). Within the PSS1-V5-miniTurbo mock group, we identified 16 proteins including Glutamate synthase 2 (FC: 2.33, *p*-value: <0.01), H^+^-ATPase 6 (FC: 2.28, *p*-value: <0.01), heat shock cognate protein 70-1 (FC: 2.23, *p*-value: <0.01), Transducin/WD40 repeat-like superfamily protein (FC: 2.14, *p*-value: <0.01), and Haloacid dehalogenase-like hydrolase (HAD) superfamily protein (FC: 2.11, *p*-value: <0.01) were enriched and are presumably PSS1-interactors (**Figures 2B**, **3**, **Table 3**).

## Discussion

In this study, we leveraged a miniTurbo-based proximity labeling approach coupled with mass spectrometry to profile the interactomes of PSS1, a glycine-rich integral plasma membrane protein with no previously known interactors. Using both wild-type PSS1 and the nonfunctional PSS1^G119D^ mutant as baits in a miniTurbo-based proximity labeling assay, we were able to identify proteins associated with PSS1. Some of these putative PSS1 interacting proteins could be involved in the expression of nonhost immunity. By fusing miniTurbo to the N- and C-termini of PSS1 and PSS1^G119D^, we facilitated *in vivo* biotinylation of proteins that transiently interact with PSS1 and PSS1^G119D^. The use of wild-type *Arabidopsis* Col-0 as a control enabled the removal of background biotinylated proteins. PSS1^G119D^, which lacks nonhost immunity function against *P. sojae* and *F. virguliforme*, served as a negative control to exclude proteins that are not involved in the nonhost immunity function. The use of these controls allowed us to reveal 17 PSS1-associated proteins following *P. sojae* infection: (i) 12 PSS1-associated proteins, when miniTurbo was fused to the N-terminus, and (ii) five when miniTurbo was fused to the C-terminus of PSS1 (**Figure 2**; **Tables 2**, **3**). AIUpred-based *in silico* structural analysis predicted that the N-terminus of PSS1 to be more intrinsically disordered than the C-terminus (Erdős and Dosztányi, 2024). This observation aligns with the outcomes of the proximity labeling study suggesting that the intrinsically disordered N-terminal fusion miniTurbo protein possesses enhanced accessibility to proximal proteins for interaction (Minde et al., 2020). This structural information may explain the differential labeling patterns seen between N- and C-terminal fusions (**Tables 2**, **3**). Disorder predictions are *in silico* and should be complemented with other orthogonal structural data, such as the ones predicted by AlphaFold models.

Biotin concentration optimization experiments revealed that the miniTurbo-V5-PSS1 fusion showed robust biotinylation at low biotin concentrations (≥ 25 µM), whereas the PSS1^G119D^ mutant required higher concentrations. This disparity may result from structural perturbations due to the glycine-to-aspartic acid substitution, which could alter protein folding or biotin ligase accessibility. An alternative explanation for the reduced labeling of PSS1^G119D^ is that the mutant protein may be intrinsically unstable and accumulate at lower steady-state levels compared to the wild-type protein. This possibility could be addressed in future studies by assessing degradation kinetics using cycloheximide chase assays or testing for proteasome-mediated turnover through inhibitor treatments. Additionally, fusion PSS1^G119D^-fluorescent protein constructs could be used to monitor its relative accumulation *in vivo*, while transcript-levels determined by RT-qPCR to determine if there is any transcriptional and post-translational regulation of the mutant gene. Together, these approaches would clarify whether the observed labeling differences stem from altered protein stability or other structural or functional consequences of the G119D substitution.

Considering nonhost immunity function of PSS1, some of the identified 17 proteins that specifically associate with PSS1 following *P. sojae* infection, but not with PSS1^G119D^ mutant (**Figure 3**), could be involved in mediating nonhost immunity function of PSS1 in Arabidopsis. We, therefore, investigated functions of some of the PSS1-associated proteins with over 1.5-fold enrichment in the transgenic lines as compared to the non-transgenic Col-0 control with *p* values ≤0.01. Two proteins, H^+^-ATPase 6 and Glutamate synthase 1, were identified in both N-terminal and C-terminal miniTurbo fusions under mock conditions, while one protein, Coproporphyrinogen III oxidase (LIN2), was detected in both N- and C-terminal fusions PSS1 proteins following *P. sojae* infection (**Tables 2**, **3**).

Proximity labeling can help infer a protein’s subcellular residence based on the subcellular locations of the interacting proteins biotinylated by the biotin ligase-fused target protein (Lee et al., 2016). Among the proteins consistently enriched in all three biological replicates with a probability value 0 or close to 0 with over 2-fold enrichment over the Col-0 background, H^+^-ATPase 6, a plasma membrane proton pump, is particularly notable. Proximity between PSS1 and H^+^-ATPase 6 was detected prior to *P. sojae* infection but this putative interaction disappeared following *P. sojae* inoculation (**Tables 2**, **3**). This observation was consistent in both N- or C-terminal miniTurbo-fused PSS1 proteins. This loss of association could reflect epitope masking, altered accessibility, or subcellular relocalization of the fusion proteins following *P. sojae* infection. Bioinformatic localization tools TargetP, SUBA5 (Hooper et al., 2022) predict plastid localization for PSS1 (**Supplementary Table 4**). On the contrary, many of the candidate PSS1-associated proteins are plastid-localized (**Tables 2**, **3**). These observations support the hypothesis that PSS1 is a dynamic protein, whose subcellular residence may have shifted in response to *P. sojae* infection.

The lack of parallelism between the PSS1 and PSS1^G119D^ associated proteins suggests that the G119D mutation might have caused significant structural changes or its instability leading to failure of the mutant PSS1 protein to associate with proteins that are involved in the nonhost immunity. Among the PSS1-associated proteins, several are implicated in plant defense. For example: PAL1, a key enzyme in the phenylpropanoid pathway, contributes to salicylic acid biosynthesis and systemic resistance. It plays a central role in responses to biotic and abiotic stress (Huang et al., 2010; Mauch-Mani and Slusarenko, 1996). HSP70-1, a molecular chaperone, has been shown to modulate PTI and ETI responses and is a known target of *Phytophthora* effectors (Lee et al., 2018; Berka et al., 2022). ATMEPCRA, a pectin methylesterase, is associated with cell wall remodeling and plant-pathogen interactions. High pectin methylesterification levels correlate with increased pathogen resistance (Lionetti et al., 2012).

LIN2/HEMF1/ATCPO-1, a coproporphyrinogen III oxidase, is involved in tetrapyrrole biosynthesis and ROS regulation. It is linked to defense-related cell death and was enriched in *P. sojae*-infected plants carrying either of the N- and C-terminal PSS1 fusion with miniTurbo. It was not detected in the mock controls (**Tables 2**, **3**), suggesting a pathogen-induced association only with PSS1, but not with the PSS1^G119D^ mutant protein (Tanaka and Tanaka, 2007; Guo et al., 2013; Madsen et al., 1993).

The observed spatial association between PSS1 and thioglucoside glucohydrolase 1 (TGG1), a myrosinase involved in glucosinolate (GLS) hydrolysis, is noteworthy given its potential role in nonhost immunity. GLSs are spatially separated from TGG1 under normal conditions. Upon tissue damage caused by pathogenic infection or pest invasion, GLSs are mobilized toward TGG1, triggering the enzymatic release of toxic volatile compounds such as isothiocyanates. These defense metabolites accumulate in guard cells and contribute to the plant’s first line of defense against pathogens that exploit stomatal openings for entry (Barth and Jander, 2006; Calmes et al., 2015; Tierens et al., 2001; Zhang K. et al., 2019; Pastorczyk and Bednarek, 2016; Bednarek et al., 2009). The enrichment of TGG1 in *P. sojae*-challenged samples, despite the absence of a clear homolog in soybean (BLASTp, Nov 2024), supports our hypothesis that PSS1 may regulate glucosinolate metabolism as part of a nonhost-specific immune response. To further test this model, we propose the following hypotheses for experimental validation: (1) assess whether PSS1–TGG1 proximity increases upon infection using proximity labeling or split-GFP approaches; (2) quantify stomatal closure and GLS-derived metabolite accumulation in PSS1-deficient lines; and (3) examine dynamic relocalization of PSS1 via time-lapse confocal microscopy during pathogen challenge.

Together, these PSS1-associated proteins highlight potential defense pathways through which PSS1 may mediate the nonhost immunity. The idea that PSS1 might modulate secondary metabolism or ROS-associated defense pathways through spatial association with multiple defense-associated proteins or enzymes is particularly intriguing. Transducin/WD40 repeat-like protein, DUF642 protein, and methylesterase PCR A involved in cell wall biosynthesis or modification were identified to be spatially associated with PSS1 when miniTurbo was fused to its N-terminus (**Table 2**) (Guerriero et al., 2015; Cruz-Valderrama et al., 2019). It appears that PSS1 most likely regulates cellulose biosynthesis and cell wall modification through three distinct proteins that controls cell wall biogenesis and modification. Therefore, it is possible that PSS1 regulates the broad-spectrum cell wall-associated immunity mechanisms for conferring nonhost immunity in Arabidopsis (Wan et al., 2021). Our findings offer novel insights into immune protein networks and provide foundational resources for dissecting nonhost immunity mechanisms in plants.

This study revealed three classes of defense-related proteins that appear to be associated more readily with PSS1 than with the G119D variant. These proteins are broadly linked to (i) glucosinolate-mediated resistance, (ii) reactive oxygen species (ROS)-associated hypersensitive cell death, and (iii) cell wall-related immune responses. While further validation is needed to confirm direct interactions, their enrichment in PSS1-specific datasets suggests potential functional connections. *P. sojae* is capable of invading individual cells of the Arabidopsis *pen1–1* mutant, which lacks a functional soluble N-ethylmaleimide-sensitive factor attachment protein receptor (SNARE), but not those of the Col-0 ecotype (**Figure 4A**; Sumit et al., 2012). SNARE proteins mediate vesicle fusion and the targeted exocytosis of antimicrobial compounds at pathogen entry sites (Collins et al., 2003). The inability of *P. sojae* to penetrate Col-0 cells (Sumit et al., 2012) suggests that the selective proximity of PSS1 but not PSS1^G119D^ to these three classes of defense-related proteins is unlikely to result from generalized protein overaccumulation in infected tissues. Instead, this pattern may reflect specific recruitment or stabilization of PSS1 at sites of immune activation. Taken together, these findings could support a functional role for PSS1 in nonhost resistance, potentially through its involvement in coordinating vesicle-mediated delivery of defense components.

Our work demonstrates the utility of miniTurbo-based proximity labeling coupled with mass spectrometry for elucidating the interactomes of a glycine rich protein involved in nonhost resistance. Three previously well characterized defense mechanisms could be regulated by PSS1 to confer nonhost immunity of Arabidopsis against an oomycete soybean pathogen. We speculate that the spatial proximity of PSS1 with these defense-related factors could have trigger positive feedback effects for rapid accumulation of defense metabolites in enhanced quantities to provide durable and broad-spectrum nonhost immunity against the soybean pathogen (Ratushny et al., 2012).

Surprisingly, the miniTurbo‑fused PSS1 was not detected among the enriched proteins in our mass spectrometry datasets. This is in contrast to detection of the baits as often the most abundant biotinylated proteins in earlier studies (Branon et al., 2018). The failed detection of any of the four miniTurbo fusion proteins could be explained by one or more of the following possibilities: (1) poor MS detectability of PSS1 peptides. (2) protein instability, degradation, or cellular toxicity. (3) lack of surface-exposed lysine residues necessary for efficient biotinylation; and (4) potential conformational change induced by miniTurbo fusion could interfere with *cis*-biotinylation (May et al., 2020; Karpievitch et al., 2009; Zhang et al., 2021). These factors may also account for the relatively modest number of proteins recovered in the N- and C-terminal datasets (475 and 270, respectively; **Table 1**).

Although miniTurbo-based proximity labeling enabled the identification of candidate PSS1-associated proteins, our approach does not confirm direct physical interactions of these proteins with PSS1. Validation of the interactions of these putative PSS1 interactors, especially those involved in production of glucosinolate and ROS and activation of cell wall-associated plant immunity mechanisms with the aid of orthogonal validation methods including co-immunoprecipitation (Co-IP), bimolecular fluorescence complementation (BiFC) and Förster resonance energy transfer (FRET) is warranted prior to establishing the role of these factors on nonhost immunity.

## Conclusion

The three classes of defense-related proteins conferring (i) glucosinolate-mediated resistance, (ii) reactive oxygen species (ROS)-associated hypersensitive cell death response and (iii) cell wall-related immune responses could interact with PSS1 following *P. sojae* infection. PSS1 could have a possible positive feedback regulation on these distinct defense mechanisms for conferring nonhost immunity (Ratushny et al., 2012). To further establish the contribution of the identified three classes of defense-related proteins on nonhost immunity, investigation of the responses of the T-DNA insertion or CRISPR-Cas9-induced knockout mutants for each of the selected candidate putative PSS1-associated factors to *P. sojae* is warranted. The factors conferring nonhost immunity could then be further investigated to determine how PSS1 influences these factors for protecting Arabidopsis from nonhost pathogens. It is also worthy to investigate the possible relocalization of PSS1 following *P. sojae* infection as suggested in this study. A careful time‑lapse confocal microscopy study using fluorescent protein–tagged PSS1 before and after P. sojae infection could be conducted to determine whether PSS1 indeed relocates to other subcellular compartments to mediate nonhost immunity.

## Data Availability

The datasets presented in this study can be found in online repositories. The names of the repository/repositories and accession number(s) can be found in the article/**Supplementary Material**.
