# Supplementary material for: Proximity labelling suggests association of the nonhost receptor PSS1 protein with enzymes of multiple defense pathways in Arabidopsis
Source: Front Plant Sci. 2026 Mar 5;16:1701640. doi: 10.3389/fpls.2025.1701640 (PMC12999863; doi:10.3389/fpls.2025.1701640)
Supplement: Supplementary file 1 [file Supplementaryfile1.pdf]

Supplementary Figures 1 – 5

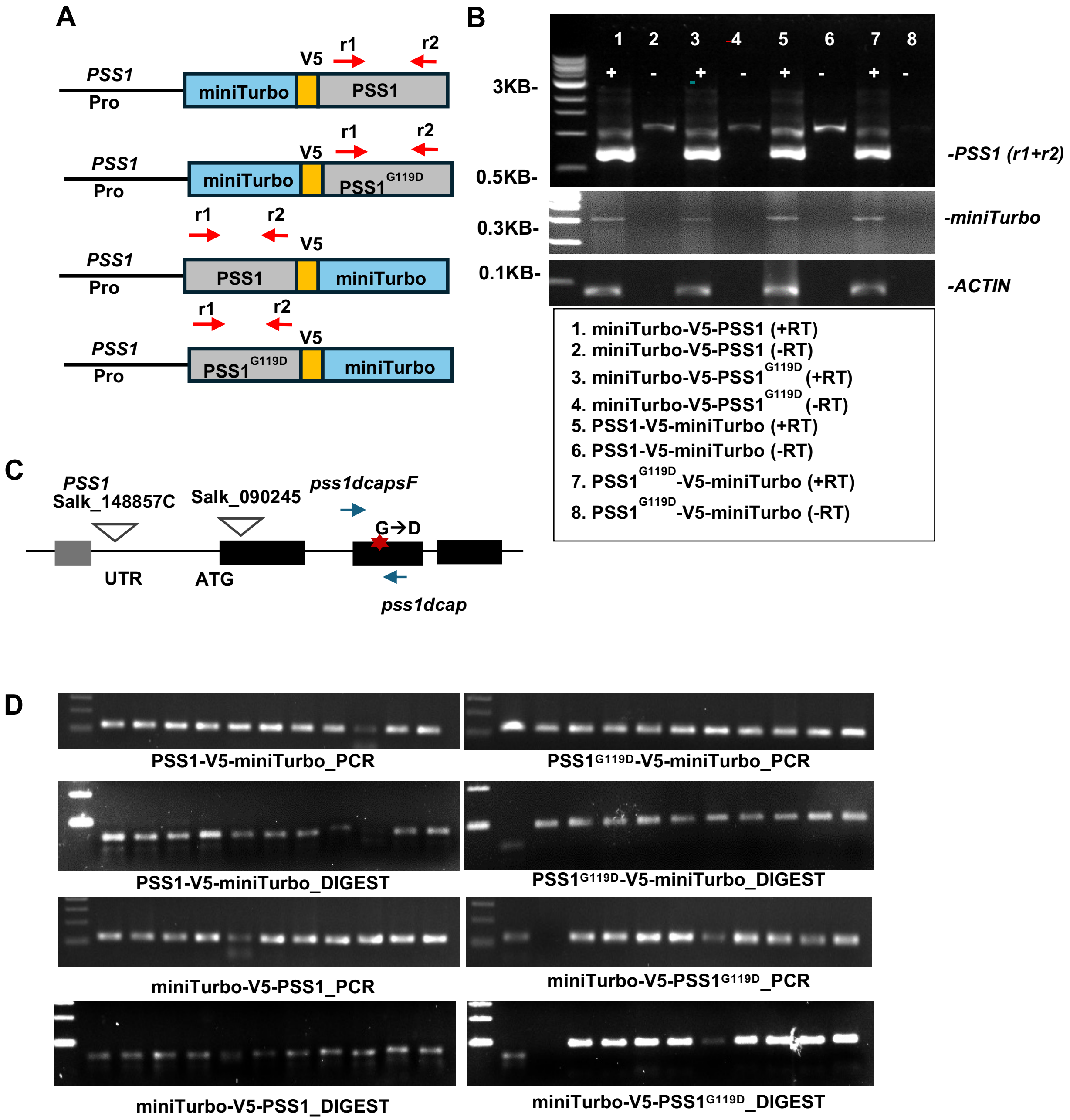

Supplementary Figure 1. Genotypic and transcript-level characterization of transgenic Arabidopsis lines expressing miniTurbo fusion constructs (supports Figure 1).

(A) Schematic representation of N- and C-terminal miniTurbo fusion constructs, showing the relative positions of primers used for reverse transcription PCR (RT-PCR).

(B) Agarose gel images showing RT-PCR amplification of miniTurbo fusion transcripts driven by the native *PSS1* promoter. Constructs were transiently expressed in *Nicotiana benthamiana* leaves via Agrobacterium-mediated infiltration. Leaf tissues were harvested 72 hours post-infiltration for total RNA extraction and cDNA synthesis. Transcript expression was assessed using construct-specific primers, with *Actin* serving as an internal control.

(C) Schematic of the second exon of *PSS1*, indicating the positions of forward and reverse primers used for genotyping.

(D) PCR-based genotyping of 10 independent T4 transgenic Arabidopsis lines per construct (miniTurbo-V5-PSS1, miniTurbo-V5-PSS1^{G119D}, PSS1-V5-miniTurbo, or PSS1$_{G119D}$ -V5-miniTurbo and Col-0 using derived cleaved amplified polymorphic sequence (dCAPS) primers (pss1dcapF and pss1dcapsR) flanking the G119D mutation site (indicated by a star). PCR products (>100 bp) were digested with *Aci*I. The G119D mutation abolishes the *Aci*I recognition site, resulting in undigested bands in mutant lines in the electrophoresis gels miniTurbo-V5-PSS1_DIGEST, miniTurbo-V5-PSS1^{G119D}_DIGES, PSS1-V5-miniTurbo_DIGEST and PSS1$_{G119D}$ -V5-miniTurbo_DIGEST. Col-0 is in Lane 1. M: DNA marker; C: wild-type Col-0 control**.**

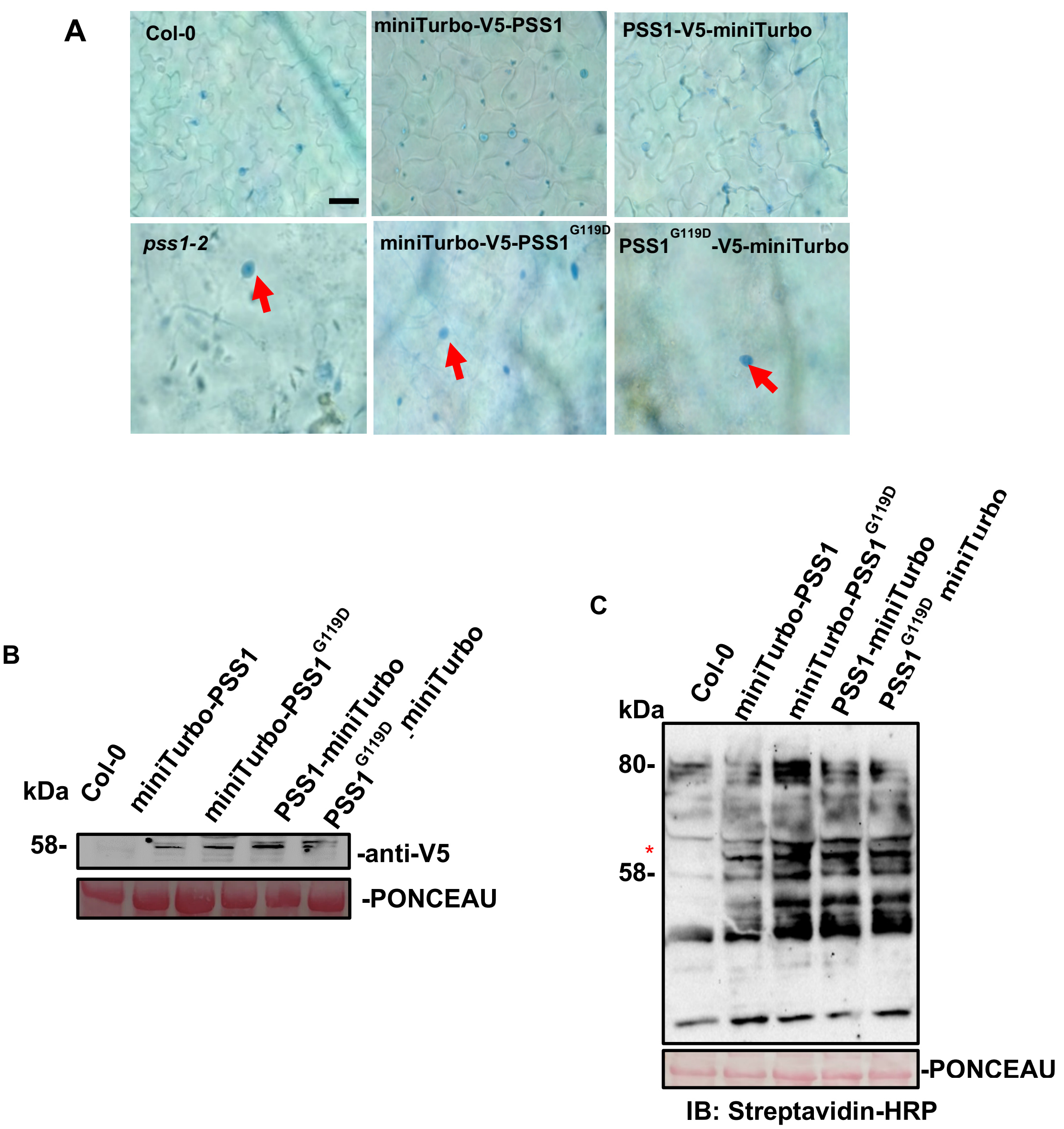

Supplementary Figure 2. Functional validation of miniTurbo fusion constructs (Supporting information for Figure 1).

(A) Trypan blue staining of leaves from 21-day-old *Arabidopsis* plants expressing miniTurbo-V5-PSS1, miniTurbo-V5-PSS1^{G119D}, PSS1-V5-miniTurbo, or PSS1$_{G119D}$ -V5-miniTurbo, Col-0 and *pss1-2* inoculated with 10 µL of *P. sojae* zoospore suspension (10^6 spores/mL) to visualize pathogen colonization. Red arrows indicate sporangia. Scale bar = 50 µm.

(B) Total protein extracts from Ten-day-old seedlings wildtype Col-0, loss of function mutants *pss1-2*, and Arabidopsis transgenic plants, miniTurbo-V5-PSS1, miniTurbo-V5-PSS1^{G119D}, PSS1-V5-miniTurbo,PSS1$_{G119D}$ -V5-miniTurbo were subjected to immunoblotting with anti-V5 antibody. Ponceau staining serves as loading control. Numbers on the side indicates the molecular weight of the proteins measured in kilodaltons.

(C) Total protein extracts from Ten-day-old seedlings wildtype Col-0, loss of function mutants *pss1-2*, and Arabidopsis transgenic plants, miniTurbo-V5-PSS1, miniTurbo-V5-PSS1^{G119D}, PSS1-V5-miniTurbo,PSS1$_{G119D}$ -V5-miniTurbo were subjected to immunoblotting with streptavidin-HRP to verify the activity of the biotin Ligase. Ponceau staining serves as loading control. Ponceau staining of the membrane is shown as loading control. Red star shows the *cis*-biotinylation of the fusion gene constructs.

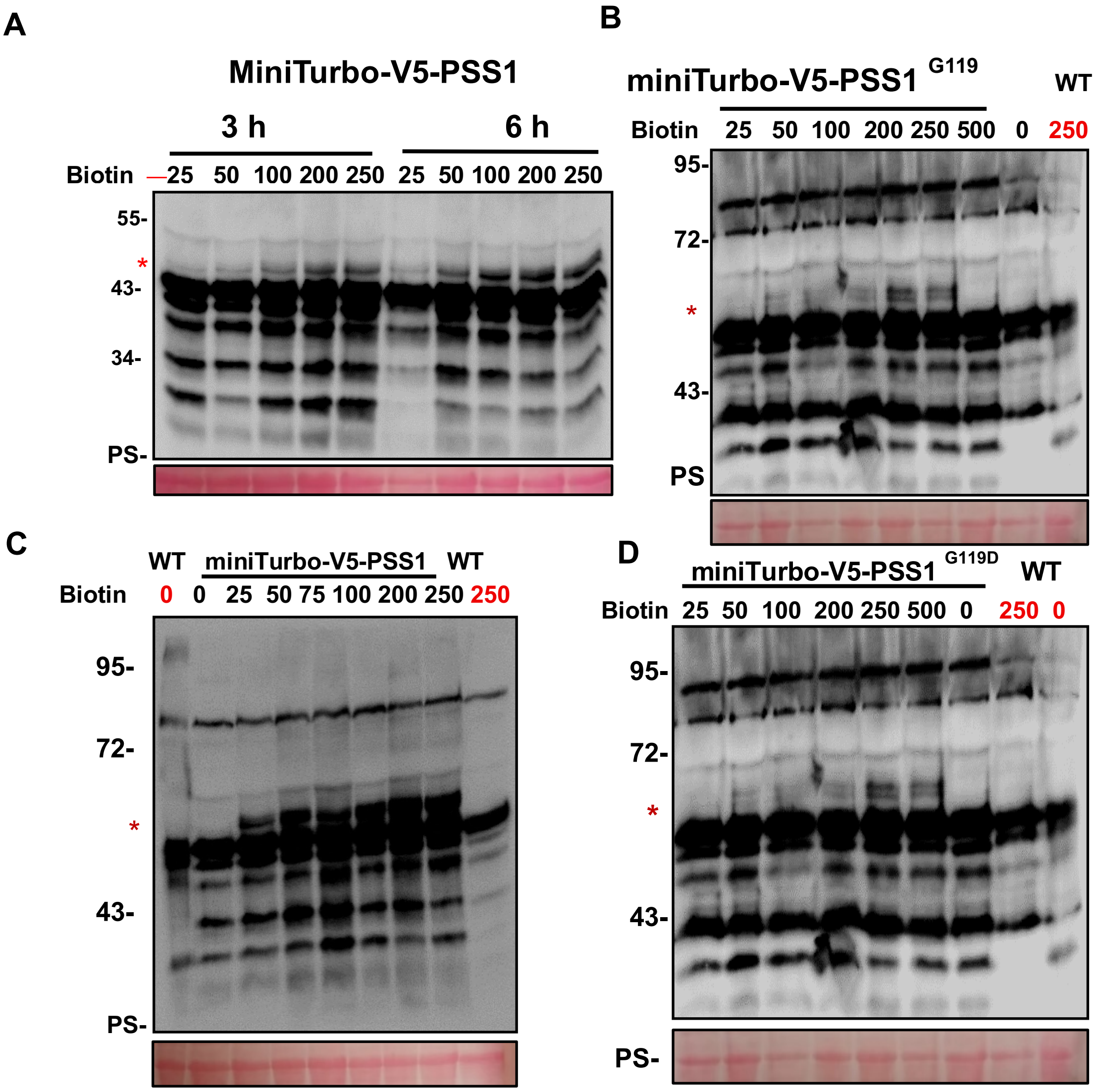

Supplementary Figure 3. Optimization of biotinylation conditions using miniTurbo-V5-PSS1 and miniTurbo-V5-PSS1^{G119D} fusion proteins.

(A) Streptavidin-HRP blots showing biotinylation levels in 10-day-old *Arabidopsis* seedlings expressing miniTurbo-V5-PSS1. Arabidopsis seedlings (miniTurbo-V5-PSS1) were grown on MS medium for 10 days and acclimatized in distilled water for 2 days followed by treatment with following treatment with increasing concentrations of biotin (25–250 µM) for 3 or 6 hours at room temperature (22 °C). The red asterisk (*) indicates the predicted cis-biotinylation of the PSS1 fusion protein. Ponceau staining served as a loading control. Molecular weights (kDa) are indicated.

(B) Streptavidin-HRP blots showing biotinylation levels in Arabidopsis seedlings expressing miniTurbo-PSS1 ^{G119D}. Arabidopsis seedlings (Col-0 (WT) and miniTurbo-V5-PSS1^{G119D}) were

grown on MS medium for 10 days and acclimatized in distilled water for 2 days followed by treatment with following treatment with increasing concentrations of biotin (0–500 µM) for 6 hours at room temperature (22 °C). WT (Col-0) in red was treated with 250 µM Biotin to serve as control. Mock control (0 µM) receive sterile distilled water under identical conditions. The red asterisk (*) indicates the predicted cis-biotinylation of the PSS1 fusion protein. . Ponceau staining served as a loading control. Molecular weights (kDa) are indicated.

(C) Streptavidin-HRP blots showing biotinylation (smear) after a 24-hour treatment. Arabidopsis seedlings (Col-0 and miniTurbo-V5-PSS1,) were grown on MS medium for 10 days and acclimatized in distilled water for 2 days followed by treatment with increasing biotin concentrations (0–250 µM) at room temperature (22 °C). WT (Col-0) was treated with 250 µM Biotin to serve as control. Mock control (0 µM) receive sterile distilled water under identical conditions. The red asterisk (*) indicates the predicted cis-biotinylation of the PSS1 fusion protein. Ponceau staining served as a loading control. Molecular weights (kDa) are indicated.

(D) Streptavidin-HRP blots showing biotinylation levels in miniTurbo-V5-PSS1 ^{G119D} seedlings. Arabidopsis seedlings (Col-0 and miniTurbo-V5-PSS1^{G119D}) were grown on MS medium for 10 days and acclimatized in distilled water for 2 days followed by treatment with increasing concentrations of biotin (0–500 µM) for 24 hours at room temperature (22 °C). Mock controls receive sterile distilled water under identical conditions. WT (Col-0) in red was treated with 0 µM and 250 µM Biotin to serve as control. Ponceau S staining (PS) of membranes served as a loading control. The red asterisk (*) indicates the predicted cis-biotinylation of the PSS1 fusion protein. Molecular weights (kDa) are indicated.

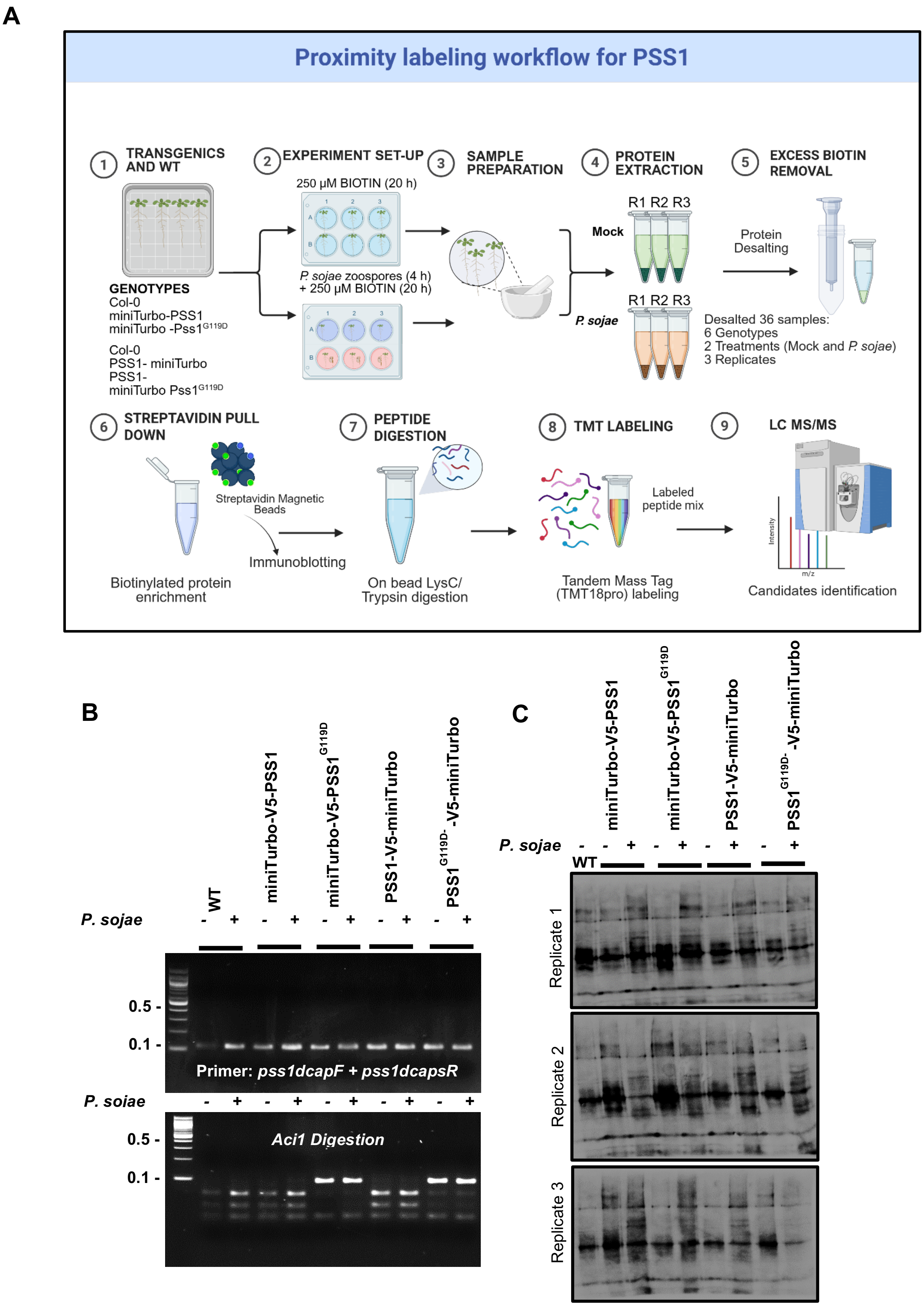
A
Proximity labeling workflow for PSS1
1 TRANSGENICS AND WT
GENOTYPES
Col-0
miniTurbo-PSS1
miniTurbo -Pss1G119D
Col-0
PSS1- miniTurbo
PSS1- miniTurbo Pss1G119D
2 EXPERIMENT SET-UP
250 µM BIOTIN (20 h)
P. sojae zoospores (4 h) + 250 µM BIOTIN (20 h)
3 SAMPLE PREPARATION
4 PROTEIN EXTRACTION
Mock
R1 R2 R3
P. sojae
R1 R2 R3
5 EXCESS BIOTIN REMOVAL
Protein Desalting
Desalted 36 samples:
6 Genotypes
2 Treatments (Mock and P. sojae)
3 Replicates
6 STREPTAVIDIN PULL DOWN
Streptavidin Magnetic Beads
Immunoblotting
Biotinylated protein enrichment
7 PEPTIDE DIGESTION
On bead LysC/ Trypsin digestion
8 TMT LABELING
Labeled peptide mix
Tandem Mass Tag (TMT18pro) labeling
9 LC MS/MS
Candidates identification
B
WT
miniTurbo-V5-PSS1
miniTurbo-V5-PSS1G119D
PSS1-V5-miniTurbo
PSS1G119D- -V5-miniTurbo
P. sojae - + - + - + - + - +
0.5 -
0.1 -
Primer: pss1dcapF + pss1dcapsR
Aci1 Digestion
C
miniTurbo-V5-PSS1
miniTurbo-V5-PSS1G119D
PSS1-V5-miniTurbo
PSS1G119D- -V5-miniTurbo
P. sojae - - + - + - + - +
WT
Replicate 1
Replicate 2
Replicate 3

Supplementary Figure 4. **Pre-mass spectrometry validation of miniTurbo transgenic samples.**

(A) Schematic overview of the experimental workflow for miniTurbo-based proximity labeling to identify the PSS1 interactomes. Arabidopsis seedlings were grown on MS medium for 10 days (1) and acclimatized in distilled water for 2 days. Twelve-day-old seedlings (wild-type Col-0, miniTurbo-V5-PSS1, and miniTurbo-V5-PSS1^{G119D}) were inoculated with *P. sojae* zoospores for 4 hours. Following infection, seedlings were treated with 250 µM biotin and incubated for an additional 20 hours in the dark at room temperature (2). For mock controls, 250 µM biotin was added without infection and incubated under identical conditions. Each sample consisted of a pooled set of 20–30 seedlings. The experiment was performed nine times and pooled into three biological replicates. Tissues from 36 samples were homogenized in liquid nitrogen for protein extraction (4) and desalting (5). Biotinylated proteins were enriched using streptavidin beads (6), digested with trypsin/LysC (7), labeled with tandem mass tags (TMT18pro), (8) and analyzed by liquid chromatography-tandem mass spectrometry (LC-MS/MS) (9). Illustration created with BioRender. (www.BioRender.com).

(B) Genotypic validation of the miniTurbo lines used for proximity labeling. Genomic DNA was extracted from transgenic seedlings and amplified using dCAPS primers (pss1dcapF and pss1dcapsR) flanking the G119D mutation site (see Supplementary Figure 1C). PCR products (>100 bp) were digested with *Aci*I and resolved on agarose gels. The presence of the G119D mutation abolished the *Aci*I restriction site, resulting in undigested PCR bands. WT: wild-type Col-0 control. Numbers on gel represent molecular ladder.

(C) Streptavidin-HRP blot confirming successful enrichment of biotinylated proteins with streptavidin magnetic beads (Step 6 in Figure S4A) prior to on-bead trypsin digestion and mass spectrometry. Three biological replicates were included per construct. Following enrichment with streptavidin beads (Step 4, Supplementary Figure 3A). 5% of the beads were boiled in loading buffer supplemented with SDS and loaded on 12% SDS-PAGE gel.

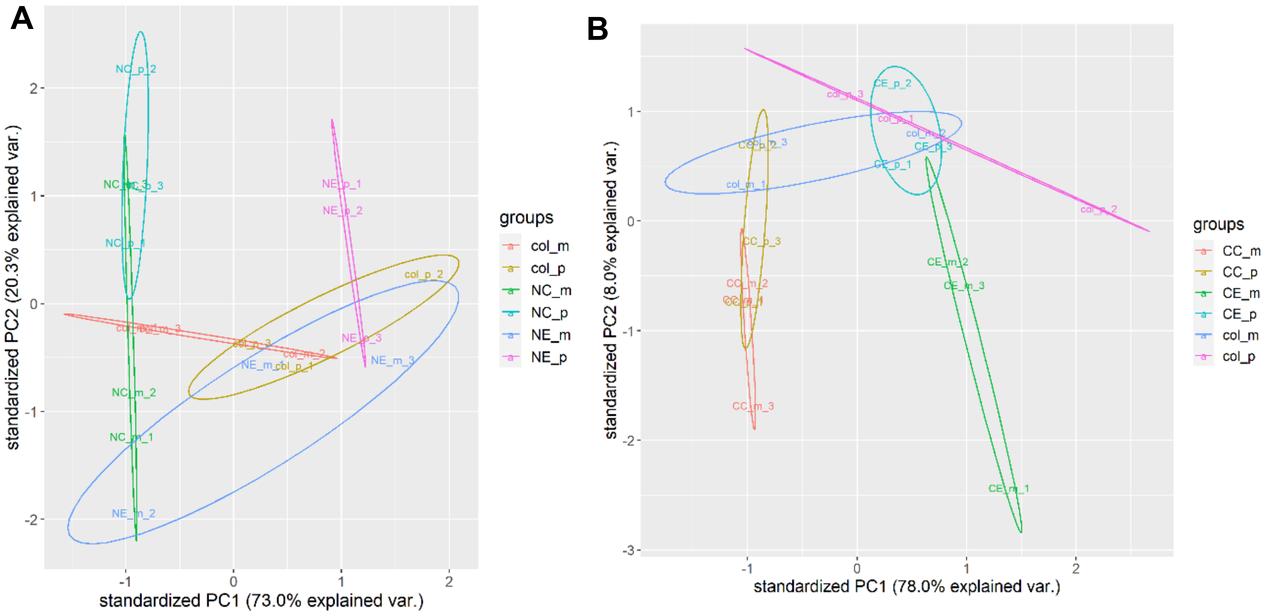

Supplementary Figure 5: Principal component analysis (PCA) of normalized mass spectrometry data from miniTurbo proximity labeling. PCA was performed using the ggbiplot R package as part of the TMT-NEAT analysis pipeline to assess global proteomic variation across genotypes and treatments. Three biological replicates (Replicates 1–3) were analyzed for each genotype.

(A) PCA plot showing interactome profiles from Arabidopsis seedlings expressing miniTurbo-V5-PSS1, miniTurbo-V5-PSS1^{G119D}, and wild-type Col-0. Samples are labeled as follows: NC_m: miniTurbo-V5-PSS1 mock-treated, NC_p: miniTurbo-V5-PSS1 infected with *P. sojae*. NE_m: miniTurbo-V5-PSS1^{G119D} mock-treated. NE_p: miniTurbo-V5-PSS1^{G119D} infected with *P. sojae*.

(B) PCA plot showing interactome profiles from seedlings expressing PSS1-V5-miniTurbo, PSS1^{G119D}-V5-miniTurbo, and Col-0 controls. Sample labels: CC_m: PSS1-V5-miniTurbo mock-treated, CC_p: PSS1-V5-miniTurbo infected with *P. sojae*, CE_m: PSS1^{G119D}-V5-miniTurbo mock-treated, CE_p: PSS1^{G119D}-V5-miniTurbo infected with P. sojae, col_m: Col-0 mock-treated, col_p: Col-0 infected with *P. sojae.*

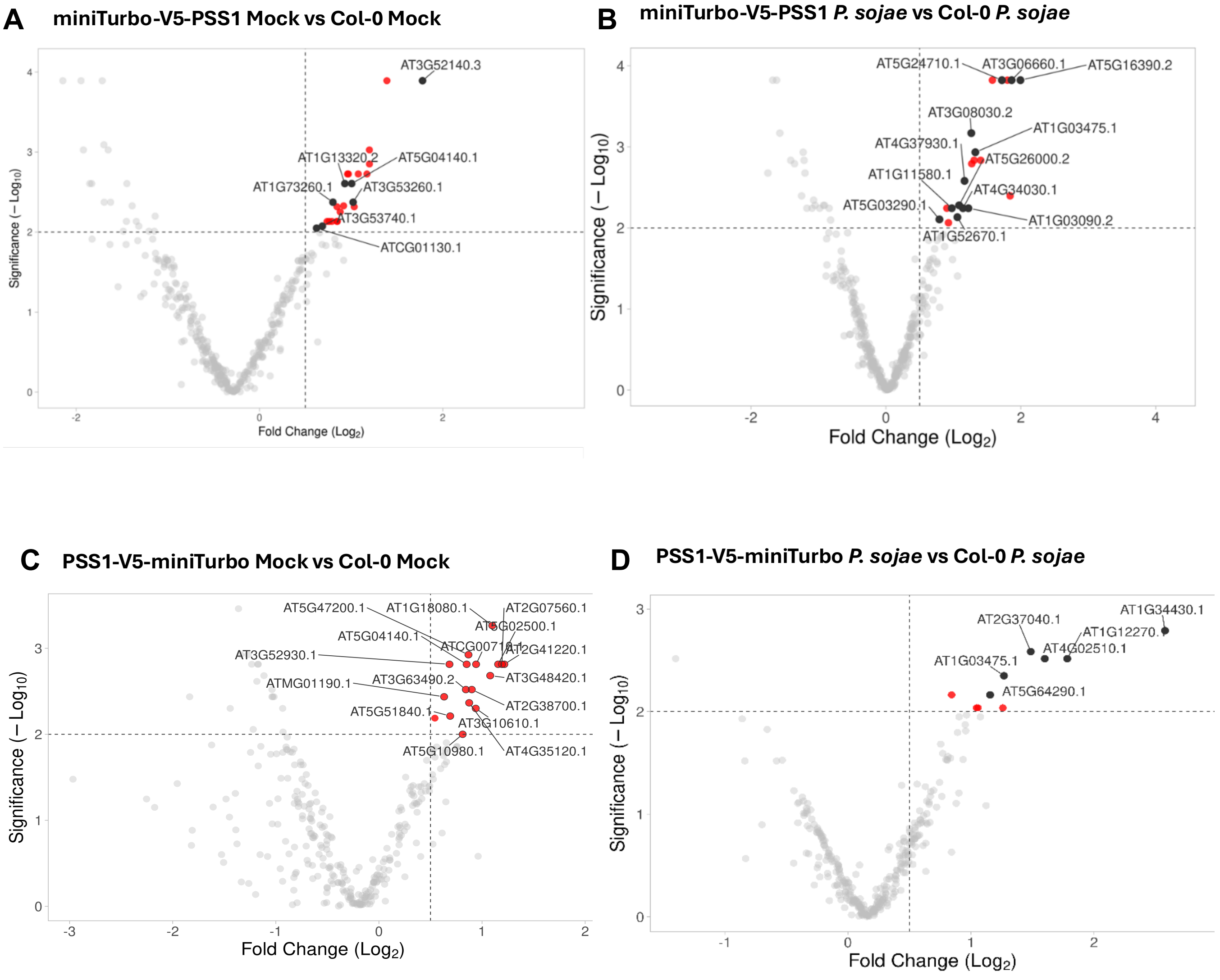

Supplementary Figure 6: Volcano plots show significantly enriched proteins in miniTurbo-V5-PSS1 and PSS1-V5-miniTurbo Mock and *P. sojae* challenged samples (Supporting information for Figure 4).

(A) Candidates enriched in miniTurbo-V5-PSS1 Mock.

(B) Candidates enriched in miniTurbo-V5-PSS1 *P. sojae.*

(C) Candidates enriched in PSS1-V5-miniTurbo Mock.

(D) Candidates enriched in PSS1-V5-miniTurbo *P. sojae*. (E) Candidates were filtered using stringent cutoffs $log_2FC > 0.5$ and log_2 pvalue > 2. Plots were generated using VolcaNoseR. Enriched proteins are labeled.

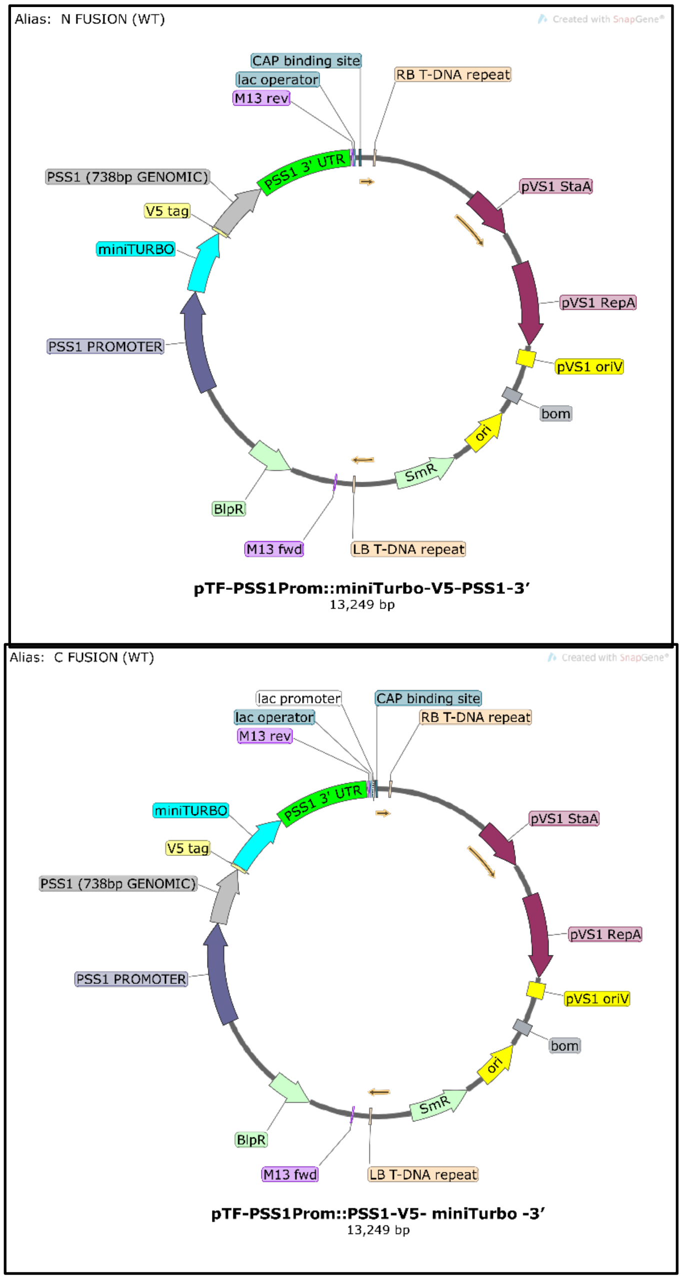

Supplementary Figure 7: Plasmid maps of binary vectors used for PSS1 expression in this study.

(A) Vector map of pTF-PSS1Prom::miniTurbo-V5-PSS1-3′, used to express wild-type PSS1 fused to miniTurbo-V5 at the N-terminus.

(B) Vector map of pTF-PSS1Prom::PSS1-V5-miniTurbo-3′, used to express wild-type PSS1 fused to V5-miniTurbo at the C-terminus. Note: Mutant versions of these vectors (pTF-PSS1^{G119D}Prom::miniTurbo-V5-PSS1-3′ and pTF-PSS1Prom::PSS1^{G119D}-V5-miniTurbo-3′) contain a glycine-to-aspartic acid substitution at position 119 (GGC→GAC).

Supplementary Tables 1 – 4

Supplementary Table 1. Primers used in this study.

PRIMER	SEQUENCE 5' to 3'	Melting Temperature in °C
V1-F	CTAGACCGGCCGAATAACTAAAATCGTCGTATTC	71
V2-R	CTAGGGGCGCGCCCCATGGGCTCATCTCTTATCTCAATG	82
V3-F	ATGAGCCCATGGGGCGCGCCCCTAGGGATCTTATGTTGATTC AAATG	82
V4-R	CCCGGGGGATCTGAAACAATTTGTTAAGATTCAAAG	72
PSS1C- F	ATGAGCCCATGGATGAGCTCTACGCAGGCTAATC	77
PSS1C- R	AGATGCGTTTGTATTTGCC	61
mTBC- F	GGCAAATACAAACGCATCTGGGAAGCCAATCCCAAAT	76
mTBC- R	GGATCCCCTAGGTTAGGACGCCTTCTCCGCTGACC	81
mTBN- F	ATGGCACCATGGATGGCAAGGGACCCCCCAGT	84
mTBN- R	GGTACTGTCAAGACCGAG	62
PSS1N- F	CTCGGTCTTGACAGTACCATGAGCTCTACGCAGGCTAA	78
PSS1N- R	GGATCCCCTAGGTCAAGATGCGTTTGTATTTGCC	75
v5mTB-Fwd	TGGAAGCGGCGGAGGTTCCGGCAAGCCCATCCCCAAC	88
v5mTB-rev	CTAGGTGCTGTCCAGGCCCAG	73
V5-Mtb-R	CTGGGGGGTCCCTTGCCATGGTACTGTCAAGACCGAG	82
mTB-F	ATGGCAAGGGACCCCCCA	73
mTB-R	TTAGGACGCCTTCTCCGC	67
Pss1rtPCRF	CGCAGGCTAATCTATGCAGACCATCCTTG	73
Pss1rtPCRR	CCAGACCAGGTAGACTTGTTGATGATC	68
mTBR-F	AGTCGCGACCATGATTCCTC	67
mTBR-R	TCAGCCATAACGATGCCTATG	64
Actin F	GGAATGGTCAAGGCAGGATTTGCTG	71
Actin R	CAATACCAGTTGTGCGACCACTTG	68
pss1dcapsF	GGGGAAGAAATCAGTTGAAG	60
pss1dcapsR	CCCATTATTCCCACCATTT	59

Supplementary Table 2. Vectors developed and used in this study.

Vector	Description	Antibiotic resistance
pbs35S::PSS1-1	Promoter, *PSS1* gene ORF and 3'UTR	Amp
pbs35S:: PSS1^{G119D}	Promoter, *PSS 1*^{G119D} gene ORF and 3'UTR	Amp
pbs35S::PSS1-2	*PSS1* gene ORF	Amp
pbs35S::pss1-1	*pss1* gene ORF	Amp
pbs35S::Prom-3'	Promoter and 3'UTR in pBlueScript	Amp
pTF101.1	Binary Vector	Spec
pTF-PSS1Prom-3′	Promoter and 3'UTR in pTF101.1	Spec
pBSDONR P4r-P2	miniTurbo-V5 plasmid	Amp
pBSV5- miniTurbo	V5- miniTurbo plasmid	Amp
pBS35S ::miniTurbo-V5-PSS1	PSS1 miniTurbo N-Fusion Construct in pBlueScript	Amp
pBS35S: miniTurbo-V5-pss1	Mutant *pss1* miniTurbo N-Fusion Construct in pBlueScript	Amp
pTF-PSS1Prom::miniTurbo-V5-PSS1-3'	PSS1 miniTurbo N-Fusion Construct in Binary Vector	Spec, Basta
pTF-PSS1Prom:miniTurbo-V5-PSS1^{G119D}-3'.	Mutant *pss1* miniTurbo N-Fusion Construct in Binary Vector	Spec, Basta
pBS35S::PSS1-V5- miniTurbo	PSS1 miniTurbo C-Fusion Construct in pBlueScript	Amp
pBS35S: pss1-V5- miniTurbo	Mutant *pss1* miniTurbo C-Fusion Construct in pBlueScript	Amp
pTF-PSS1Prom::PSS1-V5-miniTurbo -3'	PSS1 miniTurbo C-Fusion Construct in Binary Vector	Spec, Basta
pTF-PSS1Prom::pss1-V5-miniTurbo -3'	Mutant *Pss1* miniTurbo C-Fusion Construct in Binary Vector	Spec, Basta

Amp: Ampicillin, Spec: Spectinomycin, Kan: Kanamycin

Supplementary Table 3. Sample to Tandem Mass Tag (TMTpro 18-plex) label information.

**Replicate**	**Sample ID: N-Fusion Groups**	**TMT Label**	**Replicate**	**Sample ID: C-Fusion Groups**	**TMT Label**
1	miniTurbo-V5-PSS1 + Mock	126C	1	PSS1-V5- miniTurbo + Mock	126C
1	miniTurbo-V5-PSS1 + Mock	127N	1	PSS1-V5- miniTurbo + Mock	127N
1	miniTurbo-V5-PSS1 + Mock	127C	1	PSS1-V5- miniTurbo + Mock	127C
2	miniTurbo -V5- PSS1^{G119D} + Mock	128N	2	PSS1^{G119D}-V5- miniTurbo + Mock	128N
2	miniTurbo -V5- PSS1^{G119D} + Mock	128C	2	PSS1^{G119D}-V5- miniTurbo + Mock	128C
2	MiniTurbo -V5- PSS1^{G119D} + Mock	129N	2	PSS1^{G119D}-V5- miniTurbo + Mock	129N
3	miniTurbo-V5-PSS1 + *P. sojae*	129C	3	PSS1-V5- miniTurbo + *P. sojae*	129C
3	miniTurbo-V5-PSS1 + *P. sojae*	130N	3	PSS1-V5- miniTurbo + *P. sojae*	130N
3	miniTurbo-V5-PSS1 + *P. sojae*	130C	3	PSS1-V5- miniTurbo + *P. sojae*	130C
4	miniTurbo -V5- PSS1^{G119D} + *P. sojae*	131N	4	PSS1^{G119D}-V5- miniTurbo + *P. sojae*	131N
4	miniTurbo -V5- PSS1^{G119D} + *P. sojae*	131C	4	PSS1^{G119D}-V5- miniTurbo + *P. sojae*	131C
4	miniTurbo -V5- PSS1^{G119D} + *P. sojae*	132N	4	PSS1^{G119D}-V5- miniTurbo + *P. sojae*	132N
5	Col-0 + Mock	132 C	5	Col-0 + Mock	132 C
5	Col-0 + Mock	133N	5	Col-0 + Mock	133N
5	Col-0 + Mock	133C	5	Col-0 + Mock	133C
6	Col-0 + *P. sojae*	134N	6	Col-0 + *P. sojae*	134N
6	Col-0 + *P. sojae*	134C	6	Col-0 + *P. sojae*	134C
6	Col-0 + *P. sojae*	135N	6	Col-0 + *P. sojae*	135N

Supplementary Table 4: The 58 Arabidopsis proteins enriched in the purified protein samples prepared from transgenic lines carrying miniTurbo-PSS1, miniTurbo-PSS1 ^{G119D}, PSS1-miniTurbo, and PSS1^{G119D}-miniTurbo fusion proteins. A fold change > 1.5 and *p*-value < 0.01 were applied to define significantly enriched proteins in transgenic lines expressing miniTurbo-PSS1, miniTurbo-PSS1 ^{G119D}, PSS1-miniTurbo, and PSS1^{G119D}-miniTurbo fusion proteins as compared to that in corresponding wildtype nontransgenic Col-0 controls.

S.N	Gene ID	Protein ID	Location
1	AT1G03090.2	Methylcrotonyl-coa carboxylase alpha chain, mitochondrial / 3-methylcrotonyl-coa carboxylase 1 (MCCA)	Mitochondrion
2	AT1G03475.1	Coproporphyrinogen III oxidase	Plastid
3	AT1G06430.1	FTSH protease 8	Plastid
4	AT1G11580.1	Methylesterase PCR A	Extracellular
5	AT1G12270.1	Stress-inducible protein, putative	Nucleus
6	AT1G13320.2	Protein phosphatase 2A subunit A3	Cytosol
7	AT1G18080.1	Transducin/WD40 repeat-like superfamily protein	Cytosol
8	AT1G34430.1	2-oxoacid dehydrogenases acyltransferase family protein	Plastid
9	AT1G52670.1	Single hybrid motif superfamily protein	Plastid
10	AT1G73260.1	Kunitz trypsin inhibitor 1	Extracellular
11	AT1G78900.2	Vacuolar ATP synthase subunit A	Vacuole, golgi
12	AT2G01720.1	Ribophorin I	Endoplasmic reticulum
13	AT2G07560.1	H(+)-atpase 6	Plasma membrane
14	AT2G37040.1	PHE ammonia lyase 1	Peroxisome
15	AT2G38700.1	Mevalonate diphosphate decarboxylase 1	Cytosol
16	AT2G41100.2	Calcium-binding EF hand family protein	Cytosol
17	AT2G41220.1	Glutamate synthase 2	Plastid
18	AT2G47610.1	Ribosomal protein L7Ae/l30e/s12e/Gadd45 family protein	Cytosol
19	AT3G06660.1	PAPA-1-like family protein / zinc finger (HIT type) family protein	Nucleus
20	AT3G08030.2	Protein of unknown function, DUF642	Cytosol
21	AT3G09200.2	Ribosomal protein L10 family protein	Cytosol
22	AT3G10610.1	Ribosomal S17 family protein	Cytosol
23	AT3G16400.2	Nitrile specifier protein 1	Cytosol
24	AT3G17970.1	Translocon at the outer membrane of chloroplasts 64-III	Plastid
25	AT3G48420.1	Haloacid dehalogenase-like hydrolase (HAD) superfamily protein	Plastid
26	AT3G52140.3	Tetratricopeptide repeat (TPR)-containing protein	Nucleus
27	AT3G52200.1	Dihydrolipoamide acetyltransferase, long form protein	Mitochondrion
28	AT3G52930.1	Aldolase superfamily protein	Cytosol
29	AT3G53260.1	Phenylalanine ammonia-lyase 2	Peroxisome
30	AT3G53740.1	Ribosomal protein L36e family protein	Cytosol
31	AT3G55800.1	Sedoheptulose-bisphosphatase	Plastid

32	AT3G56130.1	Biotin/lipoyl attachment domain-containing protein	Plastid
33	AT3G57410.1	Villin 3	Golgi
34	AT3G63490.2	Ribosomal protein l1p/L10e family	Plastid
35	AT4G02510.1	Translocon at the outer envelope membrane of chloroplasts 159	Plastid
36	AT4G15610.1	Uncharacterised protein family (UPF0497)	Plasma membrane
37	AT4G23900.1	Nucleoside diphosphate kinase family protein	Mitochondrion
38	AT4G34030.1	3-methylcrotonyl-coa carboxylase	Mitochondrion
39	AT4G35120.1	Galactose oxidase/kelch repeat superfamily protein	Cytosol
40	AT4G37930.1	Serine transhydroxymethyltransferase 1	Mitochondrion
41	AT5G02500.1	Heat shock cognate protein 70-1	Cytosol
42	AT5G02960.1	Ribosomal protein S12/S23 family protein	Cytosol
43	AT5G03290.1	Isocitrate dehydrogenase V	Mitochondrion
44	AT5G04140.1	Glutamate synthase 1	Plastid
45	AT5G10980.1	Histone superfamily protein	Nucleus
46	AT5G16390.2	Chloroplastic acetylcoenzyme A carboxylase 1	Plastid
47	AT5G23120.1	Photosystem II stability/assembly factor, chloroplast (HCF136)	Plastid
48	AT5G24710.1	Transducin/WD40 repeat-like superfamily protein	Nucleus,plasma membrane
49	AT5G26000.2	Thioglucoside glucohydrolase 1	Vacuole
50	AT5G40770.1	Prohibitin 3	Mitochondrion
51	AT5G42080.2	Dynamin-like protein	Cytosol
52	AT5G47200.1	RAB GTPase homolog 1A	Golgi,endoplasmic reticulum
53	AT5G51840.1	Hsp90-1	Nucleus
54	AT5G63860.1	Regulator of chromosome condensation (RCC1) family protein	Nucleus,cytosol
55	ATCG00710.1	Photosystem II reaction center protein H	Plastid
56	ATCG01060.1	Iron-sulfur cluster binding	Plastid
57	ATCG01130.1	Ycf1 protein	Plastid
58	ATMG01190.1	ATP synthase subunit 1	Mitochondrion
